# Adaptive immunity to SARS-CoV-2 infection: A systematic review

**DOI:** 10.3389/fimmu.2022.1001198

**Published:** 2022-10-10

**Authors:** Marcos Jessé Abrahão Silva, Layana Rufino Ribeiro, Karla Valéria Batista Lima, Luana Nepomuceno Gondim Costa Lima

**Affiliations:** ^1^ Graduate Program in Epidemiology and Health Surveillance (PPGEVS), Bacteriology and Mycology Section (SABMI), Evandro Chagas Institute (IEC), Ananindeua, Brazil; ^2^ Bacteriology and Mycology Section (SABMI), Evandro Chagas Institute (IEC), Ananindeua, Brazil

**Keywords:** immunity, adaptive immunity, SARS-CoV-2, COVID-19, humoral immunity

## Abstract

**Background:**

There is evidence that the adaptive or acquired immune system is one of the crucial variables in differentiating the course of coronavirus disease 2019 (COVID-19), caused by severe acute respiratory syndrome coronavirus 2 (SARS-CoV-2). This work aimed to analyze the immunopathological aspects of adaptive immunity that are involved in the progression of this disease.

**Methods:**

This is a systematic review based on articles that included experimental evidence from *in vitro* assays, cohort studies, reviews, cross-sectional and case-control studies from PubMed, SciELO, MEDLINE, and Lilacs databases in English, Portuguese, or Spanish between January 2020 and July 2022.

**Results:**

Fifty-six articles were finalized for this review. CD4+ T cells were the most resolutive in the health-disease process compared with B cells and CD8+ T lymphocytes. The predominant subpopulations of T helper lymphocytes (Th) in critically ill patients are Th1, Th2, Th17 (without their main characteristics) and regulatory T cells (Treg), while in mild cases there is an influx of Th1, Th2, Th17 and follicular T helper cells (Tfh). These cells are responsible for the secretion of cytokines, including interleukin (IL) - 6, IL-4, IL-10, IL-7, IL-22, IL-21, IL-15, IL-1α, IL-23, IL-5, IL-13, IL-2, IL-17, tumor necrosis factor alpha (TNF-α), CXC motivating ligand (CXCL) 8, CXCL9 and tumor growth factor beta (TGF-β), with the abovementioned first 8 inflammatory mediators related to clinical benefits, while the others to a poor prognosis. Some CD8+ T lymphocyte markers are associated with the severity of the disease, such as human leukocyte antigen (HLA-DR) and programmed cell death protein 1 (PD-1). Among the antibodies produced by SARS-CoV-2, Immunoglobulin (Ig) A stood out due to its potent release associated with a more severe clinical form.

**Conclusions:**

It is concluded that through this study it is possible to have a brief overview of the main immunological biomarkers and their function during SARS-CoV-2 infection in particular cell types. In critically ill individuals, adaptive immunity is varied, aberrantly compromised, and late. In particular, the T-cell response is also an essential and necessary component in immunological memory and therefore should be addressed in vaccine formulation strategies.

## 1 Introduction

The 2019 coronavirus disease pandemic (COVID-19) began its occurrence around March 2020 according to the World Health Organization (WHO) through a novel coronavirus (CoV) that causes severe acute respiratory syndrome (SARS) (1). COVID-19, which arises from a *Betacoronavirus* of zoonotic origin that was first reported in Wuhan, China, has resulted in high rates of morbidity and mortality worldwide (2).

Currently, the main antigen of the virus is the SARS-CoV-2 S (Spike) protein, which can bind the virus to the human angiotensin converting enzyme 2 receptor (ACE2) through the receptor binding domain (RBD) and thus allow its entry into the cell (3–5). Clinically, patients with COVID-19 can be classified as mild (no lung involvement), moderate (with respiratory symptoms), severe (greater lung involvement, with uncontrolled systemic inflammation) and critical (requirement for mechanical ventilation and signs of septic shock) (6).

The human immune system is divided into innate and adaptive. Although innate and adaptive immune responses are interconnected and play a joint role, each of them has specialized cells with distinct functions. The adaptive system is made up of antibody-producing B cells, CD4+ T cells with various adjuvant and effector mechanisms, and CD8+ T cells with cytotoxic potential. The adaptive response is capable of producing immune memory and control of viral infection in a way that its understanding in COVID-19 is essential. However, it is proposed that the exacerbated inflammatory immune response against SARS-CoV-2 is related to the disease severity (7).

Each SARS-CoV-2 protein can initiate an immunological response that results in the generation of antibodies, and it has been shown that protein S and protein N are antigens that result in the development of neutralizing antibodies when present in high quantities (8). However, in addition to being more specific, compared to antibodies against nucleocapsid proteins, antibodies against RBD emerge sooner during the infection (9).

In patients with COVID-19, there is an incubation time of 4 to 7 days before the onset of symptoms and an additional 7 to 10 days before individual progress to severe illness. Therefore, seroconversion of the disease by IgM, although it can start on the fifth day of symptom onset, due to individual variability of the immune response, is best measured on the 10^th^ day of symptom and IgG on the 14^th^ day (10).

Both protective immunity and protection against reinfection are provided by natural SARS-CoV-2 infection. Most infected people produce IgM, IgG, and IgA antibodies against the Spike protein and its receptor-binding domain (RBD) in response to SARS-CoV-2. Although neutralizing antibodies from exposure to CoV cannot prevent infection by a variant or SARS-CoV-2, the disease can have a milder course (9, 11). Furthermore, many peptides derived from SARS-CoV-2, such as those from the M protein, induced specific memory T cell responses, as well as IFN-gamma responses, in recovered patients (12, 13).

There are signs that the immunological dysregulation caused by SARS-CoV-2 contributes more to the intensity of the disease than the virus itself. Acute respiratory distress syndrome (ARDS) and multiple organ failure are its hallmarks, while lymphocyte depletion, cytokine storm, and delayed and defective interferon (IFN) response are all present. All these factors can cause widespread lung tissue damage and subsequent thrombotic events (14, 15). It is crucial to conduct research on the virus’s defense mechanisms, especially how certain T cells interact with SARS-CoV-2, in addition to research on antibodies in the disease, is essential to fill the current knowledge gap to improve vaccine design (16).

In this situation, the question of “What immunopathological features of the virus in adaptive immunity contribute to the development of COVID-19” emerges. This study addresses this issue and develops a didactic plan for adaptive immunity from COVID-19.

## 2 Materials and methods

### 2.1 Study design

This is a systematic review of the subject with the aim of collecting concise, comprehensive, and recent data. This proposal provides an overview and visualization of the topic and can be used as a reference for future clinical trials. The research carried out the following actions: 1) Development of the central question; 2) Outline inclusion and exclusion standards; 3) Article selection; 4) Examination of articles; 5) Review interpretation, debate, and elaboration (17). The PICO technique was used for different headings to formulate the main question: Population; Intervention; Comparation; Outcome (18).

### 2.2 Search strategy and selection criteria

The search phrases used for Health Sciences Descriptors and Medical Subject Headings (DeCS/MeSH) used were “SARS-CoV-2,” “COVID-19,” “Adaptive Immunity,” “Immunity,” and “Humoral Immunity”. The descriptors and the Boolean operator “AND” were combined to search articles. The following data sources were used in the literature search: Medical Literature Analysis and Retrieval System Online (MEDLINE), Latin American and Caribbean Literature in Health Sciences (LILACS), National Library of Medicine National Institutes of Health (PUBMED), and Scientific Electronic Library Online (SCIELO).

What immunopathogenic characteristics of SARS-CoV-2 in adaptive immunity are involved during COVID-19? was the overarching topic that the studies were chosen to address. As a result, the PICOS technique generated the question, which can be understood as follows: patient population with COVID-19; the intervention to assess immunopathogenic features in SARS-CoV2 infected patients; the comparison links the virus’s immunopathogenic properties to adaptive immunity; the disease’s progression determines the outcome. Articles of the original, systematic review, clinical trial, quasi-experiment, ecological, comparative, multicenter, case series, case-control, cohort studies (prospective and retrospective), and meta-analyses types are included in study designs. Studies published between January 2020 and July 2022 that were fully available in English, Portuguese, or Spanish met the inclusion requirements. Publications published before 2020, those only accessible in the abstract, letters to the editor, and articles or materials with subjects unrelated to the study issue were excluded.

### 2.3 Study selection and data extraction

Two researchers (MJAS and LRR) separately read the titles and abstracts of the indicated papers, proceeding to full-text review of any publication judged possibly relevant by either researcher. If there was a difference of opinion, it was resolved by dialogue with another author (KVBL). The PRISMA flowchart was used and is a component of the PRISMA protocol used to illustrate the investigations in the database, document all stages, inclusions and exclusions, and demonstrate how the final sample was collected. The following information was extracted: author, title, publication year, type of study, goals, and results. Then, using Microsoft Office Excel 365, they were arranged in pairs and independently.

## 3 Results

### 3.1 Literature search

**Figure 1:**
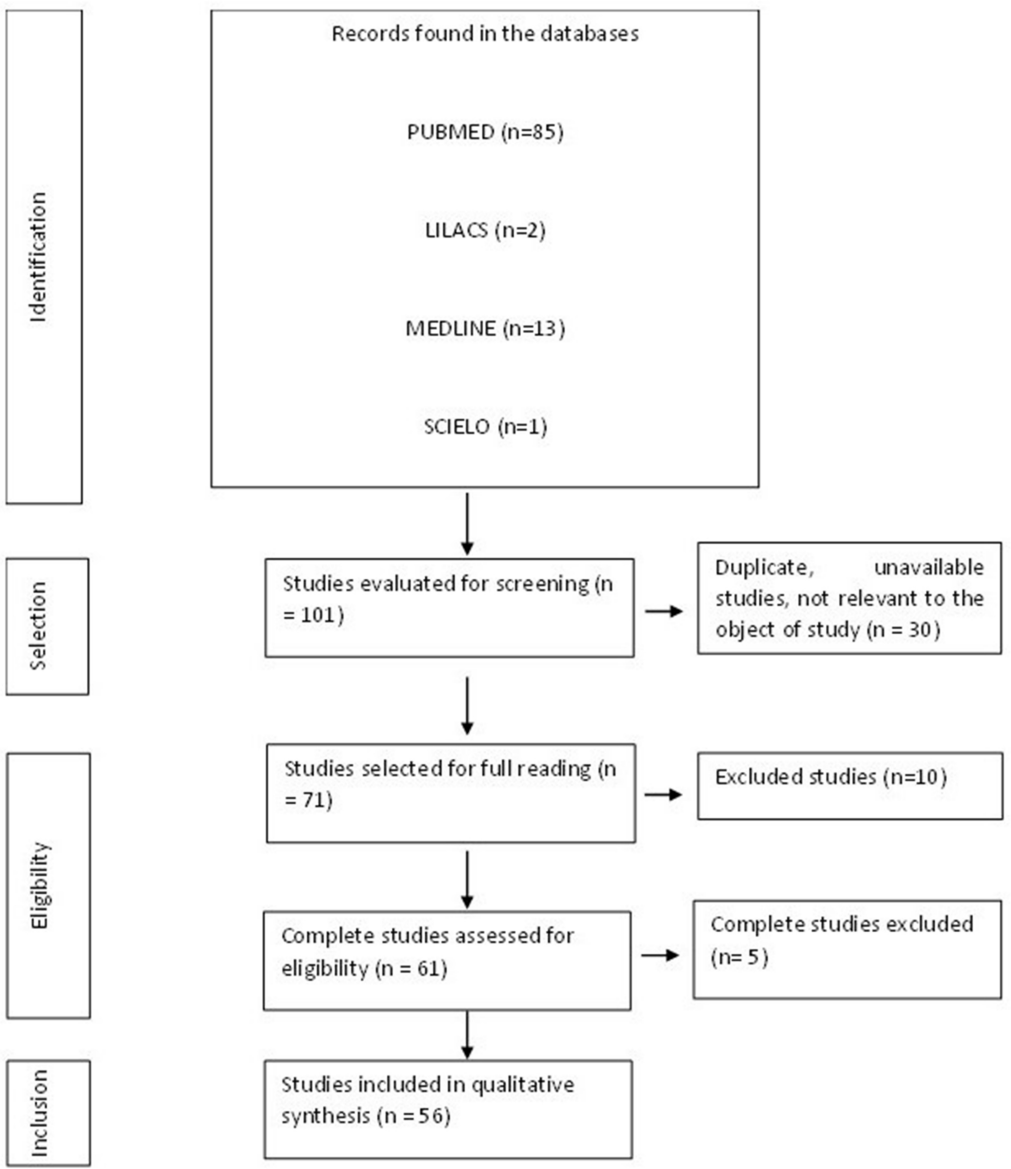
Flowchart of procedures for identification, selection, eligibility, and inclusion of studies for analysis. Belém, PA, Brazil, 2021.

When the inclusion criteria were applied, 101 articles were found, although some of them were letters to the editor, incomplete, or contained material unrelated to the study subject (**Figure 1**). A total of 56 articles formed the final structure of the articles.

### 3.2 Adaptive immune response to SARS-CoV-2 infection

As CD4+ T cells are related to the first response to infection, their percentage is higher than that of CD8+ T cells discovered after infection (7). The integrated adaptive immune response has been linked to a milder form of the disease, indicating a function of CD4+CD4 + and CD8+ T cells in the protective immunity of COVID-19 (19). A higher percentage of SARS-CoV-2-specific CD8+ T cells was observed in moderate patients (16). SARS-CoV-2-specific T cells in the acute-phase display a highly activated cytotoxic characteristic that is directly related to several clinical markers of disease intensity (20). The lungs of these seriously sick individuals also saw a large inflow of circulating T cells, overwhelming the local T cell compartment and suggesting vascular leakage. Patients in hospitals were distinguished by powerful stimulation of particularly potent or deficient CD4 + T cells and CD8 + T cells, reduced robust CD4 + T cells and CD8 + T cells comparable to highly functioning effectors, and absence of lymphocyte reaction (11).

The severity of the illness is correlated with the low IFN-γ and tumor necrosis factor – alpha (TNF-α) expression in CD4 + T cells and the elevated levels of granzyme B and perforin in CD8 + T lymphocytes. Furthermore, compared to moderate cases and healthy individuals, severe patients with COVID-19 had a significantly higher frequency of the depleted fraction (programmed cell death protein 1 [PD-1], CTLA-4, TIGIT) of CD8 + T cells. In severe COVID-19 infection, it showed reduced frequencies of CD4+ T cells producing IFN-γ. These data suggest that the poor outcome of the COVID-19 is related to functional abnormalities with respect to functional and loss of loss of CD8 + T cells (21).

A greatly increased population of CD8 + T cells was discovered in instances with moderate symptoms, indicating a link between a strong adaptive cellular immune response and improved disease management. Compared with healthy controls, CD8+ T and Natural Killer (NK cells) of COVID-19 patients expressed more inhibitory receptors of NKG2A (14). Although CD4 + T cells were not affected by SARS-CoV-2 infection, CD8 + T cells were. This response was characterized by the concurrent production of granzymes A and B, as well as perforin, in several subsets of CD8 + effector T cells. During primary infection, CD8+ T cells that expressed PD-1 also generated cytotoxic molecules, demonstrating that they were not physiologically exhausted (22). Despite a decrease in CD8+ T lymphocyte count, individuals with severe COVID-19 had a rate of HLA-DR expression in these cells that was higher than that of patients with mild disease (15).

Qualitative CD4+ T cell responses from critically ill individuals were compromised. In severe cases, Th cells begin to differentiate and develop atypically after Th0, resembling Th1, Th2 and Th17 but lacking primary functional immune features and regulatory T cells (Treg cells) (23). The different T and B cell responses shown might be a sign of an unbalanced immune response in people with COVID-19 who are seriously ill (24). Additionally, in severe cases of COVID-19, there are considerably fewer CD4+ memory T and Treg cells. Furthermore, these findings were related to a reduction in CD4+ and CD8+ T lymphocytes in the lymph nodes. Patients with COVID-19 atrophy of the spleen and lymph nodes, demonstrating the contribution of SARS-CoV-2 to the promotion of cell death. These results show that the number of CD4+ and CD8+ T lymphocytes is considerably reduced in patients with severe COVID-19 (15).

Patients with severe cases showed more robust T cell immune responses than patients with mild instances, which may indicate that patients with severe cases had better T cell memory against SARS-CoV-2. Evaluation of changes in the peripheral T and B cells of COVID-19 patients showed a negative connection between the humoral immune response and the immunological memory of T cells and the severity of the disease (13).

Follicular helper T cells (Tfh), a subset of CD4+ T cells that offer the support needed for B cell differentiation and antibody affinity maturation, promote the aging process of B cells in the restoration of COVID-19 (25). The specific IgG antibody response was unusually potent in these individuals. The tight relationship between the raised IgG response and the severity of the illness suggests a straightforward supplementary measure to distinguish between severe and nonsevere patients. In patients with COVID-19, a higher total antibody titer was independently linked to a poorer clinical outcome and increased much faster than IgM and IgG (26). In recovered patients, there was an inverse correlation between the frequency of memory B cell subsets and the severity of symptoms. However, they discovered that circulating megakaryocytes, elevated erythropoiesis, and proliferative and metabolically active plasmablasts are all increased in severe illness (27).

Early and transient rises of IgA, IgM, and, to a minor extent, IgG are related to asymptomatic SARS-CoV-2 infection. Severe illness is characterized by persistently high levels of IgA and IgG that are produced very late in the process of infection (28). IgA antibodies predominate the initial humoral responses unique to SARS-CoV-2. Shortly after the appearance of symptoms, peripheral growth of IgA plasmablasts with the ability to return to the mucosa was observed, culminating during the third week of sickness. IgG, IgM, and IgA were all viral-specific antibody responses, but IgA was more effective than IgG in neutralizing the virus (29).

The severity of the disease correlates with the increase in neutralizing titers (30). Additionally, it has been shown that the IgM or IgG nonresponder group had a considerably lower neutrophil-to-lymphocyte ratio than the IgM or IgG responder group. Patients with an IgM antibody response had substantially fewer CD20+ lymphocytes, while patients with an IgG antibody response had significantly more NK cells and CD4+ T cells than patients without an IgG antibody response. Furthermore, compared to the group, patients who developed IgM or IgG antibodies had a markedly higher proportion of total T lymphocytes and NK cells (31). Hypercoagulation and fibrinolysis can result from the deficiency or loss of hereditary angioneurotic edema function (C1-INH) (32).

In extreme instances, a reduction in RM, resting memory T cell frequency, and an increase of tissue-like memory, TLM subgroup of B cells were seen. Based on the expression of CD21 and CD27 in T cells, variations were found between subsets of B lymphocytes. S and M-specific CD4+ may impact the composition of the composition of the S1-specific B cell memory fraction (12).

The characteristics of the articles included in the formation of this review are described in the table below (**Table 1**). The articles were mostly international (n=55) and some national (n=1) derived from PUBMED, LILACS, MEDLINE, and SCIELO. The review resulted in 55 articles in English and one in Portuguese.

**Table 1:** Characteristics of the studies included in the systematic review.

Authors (References)	Kind of study	Objectives	Results
Grifoni et al., 2020 (7)	Case- control.	Measure and understand human CD4+ and CD8+ T cell responses to SARS-CoV-2 infection.	The proportion of CD4+ T cells is greater than that of CD8+ T cells found after infection, with CD4+ T cells associated with the primary response to infection. A robust CD4+ T cell response to the spike protein (S) was observed, which was correlated with anti-SARS-CoV-2 titers: IgG and immunoglobulin A (IgA). Membrane (M), S and nucleocapsid (N) proteins induced CD4+ T cell reactivity each, respectively, with 22%, 26%, and 12% of the total CD4+ response, with additional responses commonly targeting nsp3 (nonstructural protein 3), nsp4, ORF3a (open reading frame 3a) and ORF8. SARS-CoV-2-reactive CD4+ T cells were detected in approximately 40% to 60% of individuals unexposed, suggesting recognition of cross-reactive T cells between circulating ‘common cold’ and SARS-CoV-2 coronaviruses. A slightly different pattern of immunodominance was indicated for CD8 + SARS-CoV-2 T cell reactivity, with protein S accounting for ∼ 26% of the reactivity and N accounting for ∼ 12%. Significant reactivity in recovered COVID-19 individuals was derived from other antigens such as M (22%), nsp6 (15%), ORF8 (10%) and ORF3a (7%).
Rydyznski Moderbacher et al., 2020 (19)	Case-control.	Assess CD4+, CD8+ T cells and neutralizing antibody responses in SARS-CoV-2 infected individuals throughout the spectrum of severity of COVID-19 disease in a coordinated manner.	The coordinated adaptive immune response, specific for SARS-CoV-2, has been associated with milder disease, suggesting the role of CD4+ and CD8+ T cells in protective immunity in COVID-19. The coordination of antigen-specific SARS-CoV-2 responses was interrupted in subjects65 years or older. The shortage of naive T cells has also been linked to aging and poor disease outcomes.
Peng et al., 2020 (16)	Case-control	To study T cell memory in 42 patients after COVID-19 recovery (28 with mild disease and 14 with severe disease) and 16 unexposed donors, using interferon - γ -based assays with peptides encompassing SARS-CoV-2, except ORF1.	Compared to mild cases, the magnitude of T cell responses in severe cases is significantly greater. Spike specificity and total T cell response correlate with Spike-specific antibody response. 41 peptides containing CD4+ and/or CD8+ epitopes were identified, including six immunodominant regions. Six optimized CD8+ epitopes were defined, and pentamer-positive peptide MHC cells (major histocompatibility complex) showed a central and effector memory phenotype. In mild cases, a higher proportion of SARS-CoV-2-specific CD8+ T cells was observed.
Sekine et al., 2020 (20)	Cohort Study.	Systematically map the functional and phenotypic characteristics of the responses of SARS-CoV-2-specific T cells in unexposed individuals, exposed family members, and individuals with acute or convalescent COVID-19.	Acute phase SARS-CoV-2-specific T cells exhibited a highly activated cytotoxic phenotype that was correlated with several clinical markers of disease severity, while SARS-CoV-2 specific SARS-CoV-2 specific T cells were polyfunctional and exhibited memory similar to a stem cell memory T (T SCM) phenotype. SARS-CoV-2 specific T cells were detectable in family members exposed to seronegative antibodies and in convalescent individuals with a history of mild and asymptomatic COVID-19. In particular, memory CD8+ T cells from patients with acute, moderate, or severe COVID-19 expressed a different group of markers associated with activation and cell cycle, including CD38 (differentiation cluster 38), HLA-DR (human leukocyte antigen), Ki-67 and programmed cell death protein 1 (PD-1).
Oja et al., 2020 (24)	Cohort study	To characterize and quantify specific immune responses to SARS-CoV-2 in patients with different clinical courses.	Compared to subjects with mild clinical presentation, CD4+ T cell responses were qualitatively altered in critically ill patients. In these patients, the specific IgG antibody response was remarkably strong. Furthermore, in these critically ill patients, a massive influx of circulating T cells was observed in the lungs, overwhelming the local T cell compartment and indicative of vascular leakage. The disparate T and B cell responses observed may be indicative of a dysregulated immune response in critically ill patients with COVID-19.
Mateus et al., 2020 (33)	Case control	Define the repertoire of SARS-CoV-2 specific CD4+ T cells.	A range of preexisting memories of CD4+ T cells have been demonstrated to cross-react with a comparable affinity to SARS-CoV-2 and the human coronavirus common cold (HCoV) -OC43, HCoV-229E, HCoV-NL63, and HCoV-HKU1.
Yang et al., 2020 (26)	Review	Summarize the immunological features of COVID-19 and discuss the potential mechanisms of SARS-CoV-2-induced immune changes, their effects on disease outcomes, and their implications for possible treatments of COVID-19.	They reported that the percentage of memory helper T cells (CD3 +, CD4 +, CD45RO +) also decreased in severe cases compared to nonsevere cases. However, lymphopenia was present in some nonsevere and pregnant cases. Despite this, the percentage of nonsevere patients with lymphopenia are significantly lower than that of critically ill patients. Interestingly, the number of B cells is within the normal range, indicating that impaired B cells are not as significant as T cells. CD69, CD38, and CD44 have been reported to be highly expressed in CD4+ and CD8+ T cells from patients with COVID-19 compared to healthy controls. Furthermore, the expression of OX40 and 4-1BB, key molecules that promote clonal expansion and the initiation of immune responses, is markedly increased, especially in critically ill patients, indicating that T cells are likely to activate in patients with COVID-19. Activated CD4+ and CD8+ T cells have been shown to be present in the blood before symptoms subside. Additionally, T cells in patients with COVID-19 show exhaustion phenotypes. The levels of programmed cell death protein 1 (PD-1) and the immunoglobulin domain of T cells and the levels of the mucin-3 domain in CD8 + T cells are increased in overt stages of symptomatic stages compared to the prodromal stage, and S levels are detected under severe conditions. Expression of the C cell receptor subfamily member 1 is elevated in cytotoxic lymphocytes, including NK and CD8+ T cells. They found that an elevated IgG response is closely associated with disease severity, indicating a simple complementary marker to discriminate between severe and non-severe cases. A higher total antibody titer was independently associated with a worse clinical outcome in patients with COVID-19 and was significantly faster than IgM and IgG.
Tan et al., 2021 (34)	Cohort study	Investigate the dynamic changes of the virological and immunological parameters in 12 patients with acute symptomatic SARS-CoV-2 infection from the onset of the to the onset of the disease to convalescence or death.	The proportion of cells stimulated by the peptide pools covering the SARS-CoV-2 proteins remained unchanged (M (membrane), ORF3a, and Spike) or increased (N) over time. No correlation was observed when the antibody time and duration of infection were analyzed. The temporal association of the appearance of functional SARS-CoV-2-specific T cells with the reduced duration of infection suggests that T cells play an essential role in controlling SARS-CoV-2 infection. The two patients who first achieved 90% virus neutralization developed severe disease (patients P05, deceased and P03, severe). The kinetics of anti-RBD (receptor binding domain), anti-S1 (Spike S1 domain) and anti-NP IgG also peaked around the 10-20 day period, with higher peak antibody levels. against N and Spike detected in patient P05, who succumbed to infection. When the kinetics of the appearance of antibody responses against different proteins were analyzed in parallel, it was observed that patients with severe disease had an early N-polarized antibody response, while those with mild/moderate symptoms had either a dominant Spike or balanced response. It was observed that, unlike the amount of antibodies, the general magnitude of the SARS-CoV-2 peptide-reactive cells was not proportional to the severity of the disease. Although unable to directly demonstrate that CD4+ T cells were responsible for the IFN-γ response triggered by ORF7/8 pools during the early stages of infection, this interpretation was strongly supported by the unique expansion of ORF7 and ORF8 specific CD4 + T cells in convalescent patients.
Sette; Crotty, 2021 (35)	Review	Study directly adaptive immunity to SARS-CoV-2 to understand COVID-19.	The prevalence and extent of the CD4+ T cell response to SARS-CoV-2 is related to the level of expression of each SARS-CoV-2 protein. Spike, M, and N are the most significant targets for SARS-CoV-2 specific CD4+ T cells and have significant responses to ORF3a and nsp3. There is no small multichannel *crossover* with class II high affinity restricted T cells in the naive T repertoire, indicating that M is highly expressed in the body or can be used for CD4+ T cells in a very immunogenic environment. Although Spike is recognized as the most consistent SARS-CoV-2 antigen, the predicted “megapool” of class II epitopes measures approximately 50% of SARS-CoV-2-specific CD4+ T cell responses outside of Spike, making it easier to measure specific CD4+ T cells. CD4 + T cells in SARS-CoV-2 are more common in convalescent patients than in acute cases, despite the limitations of patients in Intensive Care Unit (ICU), who already have advanced conditions. SARS-CoV-2 specific CD4+ T cells had the strongest association with reducing the severity of COVID-19 disease, compared to antibodies and CD8+ T cells. They indicated that circulating IgG titers to SARS-CoV-2 are maintained for 3-4 months. Instead, a different large study by Ward et al., 2020 observed ∼ 25% of cases becoming seronegative over 6 months. Heterogeneity in viral loads and tissue distribution is present in SARS-CoV-2, in which the maximum viral load can vary >100,000-fold between cases. High titers of neutralizing antibodies are associated with severe disease and potentially extrafollicular B cell responses.
Fergie; Srivasta, 2021 (11)	Review	Summarize the understanding of immunity to SARS-CoV-2 infection, including key SARS-CoV-2 antigens (the spike protein and its receptor binding domain), its importance in vaccine development, the immediate immune response to SARS-CoV-2, the breadth of coverage of emerging variants of SARS-CoV-2, contributions of preexisting immunity to related coronaviruses, and duration of immunity.	Natural infection with SARS-CoV-2 confers protective immunity as well as protection against reinfection. The durability of immunity appears to last 3 to 5 months with SARS-CoV-2 infection. Potent neutralizing antibodies against SARS-CoV-2 appear to increase survival and may protect against reinfection with SARS-CoV-2 variants. Data suggested that immunity induced by prior exposure to CoV was insufficient to prevent subsequent SARS-CoV-2 infection, but may be associated with less severe COVID-19 through preexisting antibodies and cross-reactivity. An important role for neutralizing antibodies is antigen binding and the interaction with cells with Fc receptors to induce Fc effector functions. However, there is a potential risk of antibody-dependent enhancement (ADE) of disease mediated by these functional Fc antibodies. It was found that pregnant and non-pregnant women showed similar IgG responses recognizing the full-length SARS-CoV-2 spike protein, while pregnant women had significantly lower anti-spike-RBD IgG titers than non-pregnant women. Pregnant women were also less likely to have detectable neutralizing antibody titers. Studies of maternal serum and umbilical cord found that maternal transfer of neutralizing antibodies may be reduced, but SARS-CoV-2 infection generally did not affect maternal transfer of humoral immunity, as measured by the placental Fc receptor and antimaternal umbilical cord tetanus on IgG titers. Robust activation of highly activated or depleted CD4 + T cells and CD8 + T cells, less robust CD4 + T cells and CD8 + T cells similar to highly functional effectors, and a lack of lymphocyte response were characterized in hospitalized patients. The time to seroconversion can also predict more severe disease, which in critically ill patients was on average 4 days earlier in all isotypes (IgG, IgM, and IgA).
Maecker, 2021 (27)	Review	Understanding how the virus interacts with the host’s immune system to produce these varied results informs vaccine development.	Antibody and T cell responses are induced in most infected individuals and cross-reactive responses from other coronaviruses also exist in the uninfected population. Innate immune responses can influence the course of the disease and the character of subsequent adaptive responses. Most infected individuals produce a detectable antibody response to SARS-CoV-2, including IgM, IgG and IgA antibodies to the spike protein and its receptor-binding domain. Antibody levels usually peak at 2 weeks for IgM and 3 weeks for IgG for both nucleocapsid and protein S targets. Seropositive individuals remained seropositive for 3 to 5 months. Elevated serum levels of GM-CSF and IL-17A were correlated with more severe disease and associated with resident lymphocytes associated with Th17 cells resident in the lung. Within the lymphoid compartment, suppression of total CD3+ T cells, and particularly CD8+ T cells, has been shown to predict severe disease. Furthermore, a highly differentiated and/or exhausted CD8+ T cell compartment, which may be the result of long-term cytomegalovirus infection, can predispose to a worse outcome of COVID-19. They found that polyclonal T cells led to decreased cytokine production along with terminal differentiation in patients with COVID-19, in general. Reactive lymphocytes were also found to be lower in COVID-19 compared to other viral infections. They described a set of 12 soluble proteins, including several cytokines, whose initial level at hospital admission was predictive of mortality. Four of these (sTNFRSF1A, sST2, IL-15, and IL-10) separated survivors longitudinally during the course of hospitalization. They found an increase in activated CD8+ T cells (HLA-DR+, CD38+) along with IL-2-producing CD4+ T cells and CD4+ follicular helper T cells in severe versus mild disease. The latter can be identified by the expression of CXCR5 and PD-1. There is an inverse relationship between the frequency of memory B cell subsets and the duration of symptoms in recovered individuals. However, they found that proliferative and metabolically active plasmablasts are increased in severe disease, along with circulating megakaryocytes and increased erythropoiesis. Weak T cell responses were associated with worse outcomes in male but not female patients.
Bezemer; Garssen, 2021 (36)	Review	Map the clinical pathophysiology of COVID-19 through knowledge of virology, immunology, genomics, epidemiology, and pharmacology.	Taken together, the relatively high number of CpG motivators in SARS-CoV2 and the upstream position of TLR9 in the inflammatory cascades and the wide expression of TLR9 in different cell types play crucial roles in the clinical presentation of COVID-19 (Th1 cells, Th17 cells, B cells, neutrophils, platelets) demonstrate the great importance of TLR9 as a device that influences the risk of severe cases. TLR9 is positioned as a promising systemic therapeutic target to dampen or perhaps even prevent thrombotic complications and the so-called cytokine storm or hyperinflammatory syndrome in certain specific patients suffering from severe COVID-19.
Wang et al., 2021 (37)	Case- control.	Report specific CD4+ and CD8+ memory T cells in recovered COVID-19 patients and close contacts.	The size and quality of the memory T cell pool of COVID-19 patients has been shown to be larger and better than those of close contacts. However, the proliferation capacity, size, and quality of T cell responses in close contacts are easily distinguishable from healthy donors, suggesting that close contacts are capable of gaining T cell immunity against SARS-CoV-2 despite the lack of detectable infection. Furthermore, patients with asymptomatic and symptomatic COVID-19 contain similar levels of SARS-CoV-2-specific T cell memory.
Yang et al., 2021 (38)	Cross-sectional study	To assess whether the quantity and quality of antibodies against severe acute respiratory syndrome coronavirus 2 (SARS-CoV-2) are different between children, adolescents, and young adults.	Immunoglobulin G levels vary in different age groups, despite similar seroprevalence in pediatric and adult patient populations. SARS-CoV-2 immunoglobulin G and total antibody levels, neutralizing activity, and avidity exhibited negative correlations with age in patients aged 1 to 24 years.
Lagunas-Rangel; Chavez-Valencia, 2021 (39)	Review	Summarize the most important findings of antibody-mediated immunity against SARS-CoV-2 and highlight its participation in the efficient use of plasma from convalescent patients and the direct application of antibodies as a treatment.	Serum virus antibodies rise slightly in the early stages of the disease (the first 4 days after the onset of symptoms) and therefore may be detected in some patients (difficult to diagnose by antibody tests). Subsequently, virus-specific IgG and IgM levels in patients with COVID-19 gradually increased until the third week after the onset of symptoms, and then IgM levels began to decline, while IgG levels continued to increase, maintaining a stable antitrimeric S IgG titer of approximately three months. IgM usually peaks earlier than IgG. Virus-specific IgA is also observed in COVID-19 patients with IgM-like behavior, but it peakes on the 20th day after the appearance of symptoms. The presence of neutralizing antibodies from a previous infection was found to be associated with protection against reinfection, but at the same time, there were cases in which reinfection was considered to have occurred. The percentage of B cells and plasmablasts has been reported to be higher in patients with COVID-19 and increases with disease severity. Antibodies directed to the SARS-CoV-2 N protein showed cross-reaction with the SARS-CoV N protein, as well as antibodies directed to the SARS-CoV 2 S and S1 proteins and their SARS-CoV counterparts, but no cross-reactivity was detected. between the SARS-CoV-2 RBD protein and the SARS-CoV RBD. Approximately 97% of the patients seroconverted within 20 days of the onset of the symptom and the remaining 3% did not seroconvert, where day 12 was the median of the day for the seroconversion of IgG, IgM, and IgA. Plasma-treated patients were reported to significantly decrease viral load in the first 12 days after transfusion and improve their clinical features. Gradually, virus-specific IgG and IgM levels and their neutralizing activities increased in patients, who remained elevated for more than 7 days. As plasma cannot be produced on a large scale, the use of monoclonal neutralizing antibodies has also been suggested as a therapeutic and prophylactic strategy.
Trinité et al., 2021 (30)	Cohort Study.	To analyze the kinetics of neutralizing antibody responses and their association with disease severity.	The magnitude of neutralizing titers increased significantly with disease severity. Hospitalized subjects developed higher titers compared to mild-symptomatic and asymptomatic subjects, who together had titers below the detection limit in 50% of cases. Longitudinal analysis confirmed the strong differences in neutralization titers between nonhospitalized and hospitalized participants and showed rapid kinetics of the appearance of neutralizing antibodies (50% and 80% of maximal activity achieved after 11 and 17 days after symptom onset, respectively). in hospitalized patients. No significant impact of age, sex, or treatment on neutralizing titers was observed in this limited cohort. Longitudinal analysis confirmed the strong differences in neutralization titers between nonhospitalized and hospitalized participants and showed rapid kinetics of appearance of neutralizing antibodies (50% and 80% of maximal activity achieved after 11 and 17 days after symptom onset, respectively) in hospitalized patients.
Quast; Tarlinton, 2021 (40)	Review	Explain how B cell memory is generated by infection and vaccination, what influences its effectiveness and persistence, and how the characterization of these parameters in the immune response to severe acute respiratory syndrome coronavirus 2 (SARS-CoV-2) will help achieve immunity protection by vaccination.	T cells direct proliferation of B cells, change the class of their antigen receptor (surface bound Ab), establish a GC (germ center), and differentiate into the initial form of Ab-secreting cells called plasmablasts (PB). B cells promote the differentiation of T cells into specialized follicular T helper cells (Tfh) that, from there, direct the behavior of B cells in the immune response. Even if Ab amounts decrease over time, it seems very likely that MBCs (memory B cells) and persistent T cells will produce additional and even improved Abs upon reexposure. Protection against infection is generated through the response to infection and vaccination, which is also based on the continued or restored production of pathogen-specific neutralizing Abs. The main challenges in establishing protective immune memory for a pathogen are producing high affinity neutralizing Abs, maintaining production of such Abs, and counteracting any ongoing variability of the pathogen. The immune system has evolved to deal with these problems in memory generation, including affinity maturation in GCs, potentially lifelong survival of PCs in specialized niches, and the breadth of reactivity in MBCs.
Carsetti et al., 2020 (28)	Cohort Study.	Understand the basis of the protective immune response in COVID-19.	Early and transient increases in IgA, IgM, and, to a lesser extent, IgG are associated with asymptomatic SARS-CoV-2 infection. Persistent high levels of IgA and IgG, produced relatively late in the course of infection, characterize severe disease.
Shomura Dova et al., 2020 (41)	Case-control	Assess antibody and T cell reactivity in convalescent patients with COVID-19 and healthy donors sampled before and during the pandemic.	Healthy donors selected during the pandemic exhibited an increase in the number of SARS-CoV-2-specific T cells, but no humoral response. Their likely exposure to the virus resulted in asymptomatic infection without antibody secretion or activation of pre-existing immunity. In convalescent patients, a public and diverse T cell response to SARS-CoV-2 epitopes was observed, revealing T cell receptor (TCR) motivators with germline encoded characteristics. The extensive responses of CD4+ and CD8+ T cell to the spike protein were mediated by groups of homologous T cell receptors (TCR), some of them shared by multiple donors.
Sterlin et al., 2021 (29)	Cohort study	To measure acute humoral responses to SARS-CoV-2, including the frequency of antibody-secreting cells and the presence of SARS-CoV-2-specific neutralizing antibodies in serum, saliva and bronchoalveolar fluid of 159 patients with COVID-19.	The first specific humoral responses for SARS-CoV-2 were dominated by IgA antibodies. Peripheral expansion of IgA plasmablasts with the potential to return to the mucosa was detected shortly after the onset of symptoms and peaked during the third week of the illness. Virus-specific antibody responses included IgG, IgM, and IgA, but IgA contributed to virus neutralization to a greater extent compared to IgG. Serum-specific IgA concentrations decreased markedly 1 month after the onset of the symptom, but neutralizing IgA remained detectable in saliva longer (from 49 to 73 days after symptoms).
Kalfaoglu et al., 2021 (25)	Review	Show evidence of T-cell dysregulation in severe COVID-19 and discuss the underlying molecular mechanisms.	The response of CD4+ T cells to protein S is correlated with anti-SARS-CoV-2 IgG and IgA titers in treated patients, suggesting the importance of using T cells for B cells maturation in recovery of COVID-19, mediated by follicular helper T cells (Tfh), a type of CD4+ T cell that provides the T cell support necessary for B cell differentiation and maturation of antibody affinity. Interleukin (IL)-7 is a key cytokine for T cell homeostasis, maintaining the pool of naive T cells. However, serum levels of IL-7 increase in critically ill patients with COVID-19, indicating that the IL-7-mediated compensatory mechanism operates normally. Interestingly, the number of T cell is negatively correlated with the serum concentration of cytokines, including IL-6 and IL-10, in patients with COVID-19. IL-6 is produced mainly by macrophages, dendritic cells (DC), B cells, and T cells and can promote proliferation of T cell under inflammatory conditions. IL-10 is produced by a wide range of cells, including DCs, macrophages, B cells, and T cells, including T helper type 2 (Th2) such as IL-5 and IL-13 and regulatory T cells (Treg). IL-10 can suppress CD4 + and CD8 + T cell proliferation in some contexts while increasing T cell proliferation of T cells in the presence of other γ-chain cytokines, namely IL-2, IL-4, IL-7, and IL -15. Taking into account the increase in cytokine production in critically ill patients with COVID-19, it is unlikely that high levels of IL-10 are the cause of reduced T cells. Activated CD4+ and CD8+ T cells also increase marker expression of Ki67 proliferation markers and activation markers CD38 and HLA-DR. Since IL-2 and IL-2 receptors are increased in patients with COVID-19 that are predominantly expressed by T cells, it is possible that the positive feedback loop for IL-2 signaling is established in T cells in some patients. However, it is not known whether this is protective or contributes to the pathology of COVID-19. Patients with the disease have higher levels of the IL-6 receptor.
Carrillo et al., 2021 (9)	Review	Review current knowledge on antibodies against SARS-CoV-2, their implications for our understanding of the pathogenesis of the disease, and the development of antibody-based therapies and diagnostic tools or vaccines.	The peak antibody response between the second and third weeks after infection subsequently decreases and is characterized by the presence of IgA, IgM, and IgG in plasma and saliva. Antibodies to RBD appear earlier in the course of infection than antibodies to protein N. In addition, anti-RBD antibodies may provide greater sensitivity and specificity for diagnosis than anti-nucleocapsid responses. The Seroconversion to RBD in patients is frequent and rapid, with low cross-reactivity with SARS-CoV. Administering convalescent plasma to COVID19+ individuals improves their clinical status, at least in those treated with high neutralizing plasma during the early stages of the disease. In addition to their neutralizing activity, antibodies develop additional functions depending on their isotype. Despite the contrast in time information from protective immunity of virus antibodies, understanding of the interaction between SASR-CoV-2 and the immune system has paved the way for extraordinarily rapid vaccine development and the design of clinically tested mAbs as new therapeutic tools.
Bordallo et al., 2020 (14)	Review	Address a theoretical model on immunopathogenesis associated with severe COVID-19, based on the current literature on SARS-CoV-2 and other epidemic pathogenic coronaviruses such as SARS and MERS.	The role of CD8+ T cells in the immune response to coronavirus was highlighted in an analysis of bronchoalveolar lavage fluid (BALF) from patients with COVID-19. In mild symptomatic cases, a highly expanded population of CD8+ T cells was found, suggesting that a robust adaptive cellular immune response was related to better disease control. CD8+ T and NK cells from COVID-19 patients increased the inhibitory expression of the NKG2A receptor compared to healthy controls. This altered expression is normalized in convalescent patients. The genome-wide transcriptional signature of depleted CD8+ T cells showed altered expression of inhibitory and costimulatory receptors such as PD-1 and LAG-3. In this light, immune checkpoint inhibitors can restore effector functions and improve clearance.
Westmeier et al., 2020 (22)	Cohort Study.	To analyze the differentiation and cytotoxic profile of T cells in 30 cases of mild COVID-19 during acute infection.	SARS-CoV-2 infection induced a cytotoxic response of CD8 + T cells, but not CD4 + T cells, characterized by simultaneous production of granzyme A and B, as well as perforin, in different subsets of CD8 + effector T cells. CD8+ T cells expressing PD-1 also produced cytotoxic molecules during acute infection, indicating that they were not functionally depleted. However, in patients with COVID-19 older than 80 years, the potential for cytotoxic T cells decreased, especially in effector memory and terminally differentiated CD8+ effector cells, showing that elderly patients have impaired cellular immunity against SARS-CoV-2.
Zhang et al., 2020 (13)	Cohort study	To profile the adaptive immune cells of PBMC from recovered patients with COVID-19 with varying disease severity using single cell RNA and TCR/BCR V(D)J sequencing.	Different antibody profiles were observed among critically ill, mild patients and healthy controls, such as those related to the expansion of the clonal T cell receptor (TCR) and the B cell receptor (BCR), the isotypic distribution of antibodies, the preference for the use of segments of the V(D)J genes, and dysregulation in peripheral blood lymphocyte levels. A concomitant CD8+ and CD4+ T cell response was detected in the adaptive immune system of COVID-19 patients, and clonally expanded CD8+ effector cells in patients can ultimately develop into long-term memory T cells. Severe cases of patients exhibited stronger immune responses from T cell than mild cases, suggesting that severe cases may retain a stronger and stronger memory of T cell memory against SARS-CoV-2 than the other group of patients. Characterization of variations in peripheral T and B cells from COVID-19 patients revealed a negative correlation of humoral immune response and immune memory of T cells with disease severity, that is, regulated immune pathways in convalescent patients remain active even after infection.
Wei et al., 2020 (42)	Case study	Compare two patients with severe COVID-19 pneumonia in humoral or cell-mediated immunodeficiency states.	In the first patient in the study, plasma and B-cell dysplasia occurred long after treatment with CAR T cells and persistent CAR T cells. The SARS-CoV-2 virus successfully established infection. This patient was unable to clear the virus with a normal host immune response and was unable to produce antibodies against SARS-CoV-2, suggesting that a humoral immune response is necessary to remove the virus. In later stages, the inflammatory response is the main cause of lung damage and subsequent death. Similar to patient 1, hyperactivation of highly cytotoxic T cells such as CD8+ T cells and/or macrophages in diffuse lung tissue is responsible for severe immune injury, important in patients with COVID-19. In critically ill patients, excessive inflammation related to cytokines and damage to the lungs can cause rapidly developing pneumonia. Also in patient 1, the use of corticosteroids prevented high plasma levels of IL-6, IL-2R, and TNF-α. The consistently elevated etiology of ferritin associated with an increased risk of adverse outcomes in hospitalized patients with influenza infection has not yet been reported in the COVID-19 study. The underlying pathophysiology of elevated ferritin levels is not fully understood, but it may indicate the consequences of an underlying cytokine storm or activated macrophage system. In current clinical practice, the prognosis for HIV patients co-infected with COVID -19 and therefore with some degree of CD4+ T cell dysfunction is generally good, rarely progressing to a severe form. In case 2, long-term oral administration of a calcineurin inhibitor (cyclosporine) after kidney transplantation blocked key signaling pathways that regulate T cell activation. In patient 2, it may be related to inhibition of general immunity and specific inhibition of T cell responses. Furthermore, the humoral immunity of patients considered partially affected by cyclosporine contributed to the early elimination of the virus. Serological tests showed relatively lower titers compared to COVID-19 patients, with no effect on immune function.
Lotfi; Kalmarzi; Roghani, 2021 (15)	Review	Summarize current knowledge on the role of immune responses against SARS-CoV-2 and describe immune evasion strategies for this virus. In addition to achieving a deep understanding of the pathological immune responses involved in the pathogenesis of COVID-19, increasing the understanding of its progression to the severe form will help to identify and rationalize the design of effective therapies.	In a murine model, but not in CD8+ T cells, the evacuation of CD4+ T cells is associated with decreased lymphocyte recruitment to the lung, as well as reduced generation of cytokines and antibodies, events that cause severe immune-mediated pneumonitis and delayed clearance. of SARS-CoV-2 from the lungs. The number of CD8+ T cells decreases when infected with COVID-19. Furthermore, in severe cases of COVID-19, the number of CD4+ memory T cells and regulatory T cells (Treg cells) is significantly reduced. Furthermore, these observations were associated with a decrease in the number of CD4+ and CD8+ T cells in the lymph nodes. The spleen and lymph nodes have been described with atrophy in patients with COVID-19, highlighting the role of SARS-CoV-2 in the promotion of cell destruction. Consistent with these findings, in severe cases of COVID-19, the number of CD4+ and CD8+ T cells is significantly reduced. Although the number of CD8+ T lymphocytes was reduced, the rate of HLA-DR expression in these cells was about 35% higher in severe cases of COVID-19 than in patients with moderate disease.
Zheng et al., 2020a (21)	Cross-sectional study	To provide immunological characteristics of peripheral blood leukocytes of 16 patients admitted to the Yunnan Provincial Hospital for Infectious Diseases, Kunming, China.	They showed that low expressions of IFN-γ and TNF-α in CD4 + T lymphocytes, as well as elevated levels of granzyme B and perforin in CD8 +, are associated with the severity of the disease. Furthermore, the frequency of the CD8 + T cells (PD-1 + CTLA-4 + TIGIT +) was notably higher in severe cases of cases of COVID-19 compared to mild cases and healthy people. It revealed lower frequencies of CD4+ T cells generating IFN-γ in severe cases of COVID-19 infection. These findings indicate that CD4+ T cell functional defects and CD8+ T cell depletion are associated with a severe outcome of the COVID-19 disease.
Tavukcu Oglu et al., 2021 (43)	Case- control	To assess functional responsiveness (potency of action capacity) of CD4+ or CD8+ memory *naive*, effector, central memory, and effector T cells, which were obtained from patients with a history of COVID-19, against monocyte-derived dendritic cells (DC) carrying the SARS-CoV-2 S1 antigen.	Naive populations of T cells or effectors CD4 + CM T cells and CD8 + EM T cells exhibited the highest proliferative activity. CD4+ memory T cell proliferation can be induced in ~90% of COVID-19 patients; however, CD8+ memory T cell proliferation was only evidenced in ~60% of patients. Furthermore, surface expression of several activation-related markers, especially CD25, PD-1, and 4-1BB, was significantly increased in S1-sensitive T cells. S1 stimulated EM T cells stimulated with DC S1 secrete the highest levels of IFN-γ and IL6, most of which are produced by type 1 CD4+ T cells and cytotoxic CD8+ T cells. The cytotoxic response markers granzyme A, granuricin, and Fas ligand (FasL), especially granzyme B, were also elevated in cultures containing EM T cells from patients with COVID-19. The coculture supernatant, in which soluble factors are measured, contains mediators secreted by T cells and DC. Therefore, as shown in the literature, DC can also produce moderate amounts of cytokines, such as TNF-α, I-L6, IL-4, IFN-γ can also be produced by DC. In some cases, memoryless T cells respond to DC S1, which up-regulates the expression of CD38 and 4-1BB and secretes IL-4 and TNF-α. Furthermore, heterogeneity was observed between the parameters of the T cells studied and the time of subsequent blood collection, healing, the severity of clinical symptoms, or levels of anti-S1 antibodies.
Schöllhorn et al., 2021 (44)	Case- control	To describe a modified assay protocol for sensitive detection of specific CD4+ T cells for functional antigens using a monoclonal antibody (m24 Ab clone) specific for the open high-affinity conformation of β 2 -integrin.	The kinetics of integrin activation were different in CD4 + and CD8 + T cells (several hours and a few minutes, respectively); however, m24 Ab easily stained both cell types 4-6 h after antigen stimulation. When costing β 2 -integrin with m24 and CD154 Abs, extremely low frequencies of polyfunctional CD4+ T cells were evaluated. Antigen-specific CD4+ T cells can best be visualized with an Ab specific for activated β 2 integrins. Antigen-specific β 2 - integrins in CD8 + T cells can be visualized by ICAM-1 multimers or Ab m24 staining. Binding of m24 Ab identifies CD4+ T cells specific for functional antigen specific CD4 +. The addition of overlapping peptides derived from membrane (M), (N) or spike (S) proteins induced a clear CD4+ T cell response in previously exposed subjects, detectable by costaining with m24 and CD154 Ab, even at very low frequencies.
Rockstroh et al., 2021 (45)	Cohort study	Analyze neutralizing antibodies and specific IgG responses to different antigens, including entire *virion* inactivated entire SARS-CoV-2, Spike subunit 1 protein and its receptor binding domain, as well as the nucleocapsid protein.	Data indicate that neutralizing antibody titers, as well as antibodies that bind to total virion proteins, S1-, RBD- and nucleocapsid, are higher in early convalescent sera from severely symptomatic patients with COVID-19compared to patients with mild symptoms. During 6–9 months after the onset of symptoms (PSO), the mean neutralizing antibody titers increased in the severe group, although the difference for the mild symptoms group was statistically insignificant. Neutralizing antibodies decreased in 13% of patients with mild symptoms below the detection limit, while in all severely affected individuals, neutralizing antibodies remained detectable. Despite lower mean neutralizing titers compared to severe outcomes at the beginning of convalescence, patients with mild symptoms had a relatively wide titer range and relatively slow decay of neutralizing antibodies over time. Neutralizing antibody responses have been shown to be very individual and diverse, especially in mild to moderately symptomatic patients.
Pušnik et al., 2021 (12)	Cohort study	Investigate SARS-CoV-2 S protein-specific memory B cells and how their dependence on CD4+ T cells helps in different settings of coronavirus disease 2019 (COVID-19).	Compared to severely ill individuals, those who recovered from mild COVID-19 developed fewer functionally superior S-specific memory B cells. The generation and maturation of these cells is best associated with CD4+ T cells that express IL-21+ in recovered individuals and CD4+ T cells that express CD40L+ in critically ill individuals. On the other hand, in critically ill individuals, CD40L expression was positively correlated with IgG avidity but at the same time contributed to the activation and exhaustion of memory B cells. The increased activation and exhaustion of memory B cells observed during COVID-19 correlate with CD4+ T cell functions. Interestingly, CD4+ T cells that recognize the membrane protein show a stronger association with S-specific memory B cells than those that recognize S or nucleocapsid proteins. The greatest magnitude of SARS-CoV-2-specific CD4+ T cells was shown to be observed in critically ill individuals, with the exception of IL-4/13 secreting cells, which were more frequent in recovered individuals. The frequency of CD4+ T was also highly dependent on its specificity. In most cases, the higher response was specific for the M protein compared to those of S and N. The same trend was previously observed for the IFN-γ response for SARS-CoV-2. In addition, the functional composition of M-specific CD4+ T cells was more diverse. A decrease in the RM frequency and an expansion of the TLM subset were observed. Differences between B lymphocyte subgroups were made based on CD21 and CD27 expression (AM, CD27 + CD21 -; RM, CD27 + CD21 +; TLM, CD27 - CD21 -; IM, CD27 - CD21 +). The composition of the S1-specific B cell memory subset was potentially influenced by S and M-specific CD4+.
Lee and Oh, 2021 (46)	Review	Present important findings on humoral immune responses in COVID-19, including the immune dynamics of antibody responses and correlations with disease severity, neutralizing antibodies and their cross-reactivity, how long antibody responses and memory B cells last, aberrant antibodies and autoreactive agents generated in patients with COVID-19.	Anti-nucleocapsid protein (NCP) IgM and IgG were shown to start on day 7 and day 10 after the onset of the symptom and peak on day 28 and day 49, respectively. Furthermore, these antibodies appear earlier and their titers are significantly higher in critically ill patients than in non-severe patients. Patients with weak IgG responses were found to have a significantly higher rate of viral shedding compared to people with stronger responses, indicating that a stronger antibody response is associated with delayed viral shedding and disease severity. It revealed that S-specific humoral responses are elevated among convalescent individuals, while functional antibody responses to the nucleocapsid are increased in deceased individuals. S-specific phagocytic and complement-fixing activities were enriched in early convalescents, indicating that these Spike-specific humoral responses may be beneficial for the path of SARS-CoV-2 infection. The longevity of antibodies against SARS-CoV-2 is still unclear, but SARS-CoV-2-specific memory B cells persist for 3-6 months. Cross-reactivity has been reported between neutralizing antibodies against SARS-CoV-2 and other types of CoV. They showed that patients with COVID-19, especially individuals with severe symptoms, have a higher prevalence of autoantibodies against various host proteins.
Verkerke et al., 2021 (47)	Case- control	Define anti-SARS-CoV-2 IgA antibody levels in PC donors (convalescent plasma) and hospitalized patients.	Increased levels of anti-SARS-CoV-2 IgA antibodies were correlated with clinical improvement and viral clearance in a child with COVID-19, prompting a broader examination of IgA levels among CP donors and hospitalized patients. Significant heterogeneity in IgA levels was observed among CP donors, which was poorly correlated with IgG levels, or the results of a serological test commonly used. Unlike IgG and IgM, IgA levels were also more likely to be variable in hospitalized patients, and this variability persisted in some patients >14 days after the onset of the symptom. IgA levels were also less likely to be maintained than IgG levels after subsequent PC donation.
Cao et al., 2021 (48)	Clinical trial	Report the humoral immune response to circulating variants of SARS-CoV-2, such as 501Y.V2 (B.1.351), plasma and neutralizing antibodies (NAb) generated by CoronaVac (inactivated vaccine) ZF2001 (RBD subunit vaccine) and natural infection.	Among the 86 potent neutralizing antibodies (NAbs) identified by high-throughput single-cell VDJ sequencing of peripheral blood mononuclear cells from vaccinated and convalescents, almost half of the anti-RBD NAbs showed large reductions in neutralization against the combination of K417N/E484K/N501Y mutation, with E484K being the dominant cause. Recurrent antibodies to VH3-53/VH3-66 respond differently to variants of RBD, and K417N compromises the most neutralizing activity through reduced polar contacts with regions determining complementarity. In contrast, deletion of 242-244 (242-244Δ) deletion would eliminate most of the neutralizing activity of NAbs. Anti-NTD disrupts the conformation of antigenic supersite of NTD, indicating a much lower diversity of NAbs anti-NTD than NAbs anti-RBD. Plasma from convalescents and CoronaVac vaccinates exhibited comparable reductions in neutralization against pseudo and authentic variants of 501Y.V2, caused primarily by E484K/N501Y and 242–244Δ, with additive effects. It is important to note that RBD subunit vaccinates exhibit markedly greater tolerance to 501Y.V2 than convalescents, as the anti-RBD NAbs elicited exhibits high diversity and are not affected by NTD mutations. Furthermore, an extended interval between the third and second doses of ZF2001 leads to better neutralizing activity and tolerance to 501Y.V2 than the administration of three standard doses.
Renner et al., 2021 (49)	Case-control	Describe polyclonal T cell reactivity in patients with COVID-19 and controls.	Impaired T cell reactivity was found to be already evident in mild disease, caused by plasma components present in COVID-19 patients and strongly associated with disease severity and prolonged viral replication. Finally, a score was developed to predict the fatal outcome to identify patients who may benefit from strategies to overcome T cell hyporeactivity. A clear hyporeactivity of the hyporeactivity of T cells has been reported in hospitalized patients with COVID-19 that is pronounced in ventilated patients, associated with prolonged persistence of the virus, and reversible with clinical recovery. COVID-19-induced T cell hyporeactivity is extrinsic to T cells and is caused by plasma components, regardless of patients’ occasional immunosuppressive medication. Monocytes respond more strongly in men than in women and IL-2 partially restores T cell activation. Downstream markers of T cell hyporeactivity are also visible in fresh blood samples from ventilated patients. Gender-specific differences were observed in the downstream effects induced by T cells in monocytes. Gender-specific analysis showed that T-cell-induced up-regulation of CD123 in monocytes was significantly greater in healthy men and unventilated men than in the same groups of women. As IL-3 contributes to monocyte activation, stronger binding of T-monocytes in men may explain the recently described higher plasma levels of IL-8 and IL-18 in male patients with COVID-19.
Rezaei et al., 2021 (31)	Cross-sectional study	Assess the immune cell profile of patients with and without COVID-19with antibody response.	Leukocyte, neutrophil, and lymphocyte counts consistently decreased in the IgM and IgG non-responder group, while differences in mean value between the two study groups were statistically significant only in terms of neutrophil counts. Additionally, it was observed that the neutrophil to lymphocyte ratio was significantly lower in the IgM or IgG non-responder group compared to the IgM or IgG responder group. Patients with an IgM antibody response had significantly lower CD20+ lymphocytes, percentages of NK cells, and CD4+ T cells increased significantly in patients with an IgG antibody response compared to those without an IgG antibody response. Furthermore, patients who produced IgM or IgG antibodies had significantly higher percentages of total T lymphocytes and NK cells compared to the group without a serologic response. No significant differences were observed in the percentage of other subsets of lymphocytes, including CD8+ T cells, Treg cells, and CD19+ B cells.
Martonik et al., 2021 (23)	Review	Focus on the Th17 pathway in the course of the immune response in COVID-19 and explore plausible targets for therapeutic intervention.	Data suggest that Th17 cells play an important role in the pathogenesis of COVID-19, not only activating the cytokine cascade, but also inducing Th2 responses, inhibiting Th1 differentiation, and suppressing Treg cells. Many cytokines contribute to the differentiation of Th17 cells. Naive CD4+ cells differentiate into Th17 under synergistic exposure to TGF-β and IL-6. Furthermore, IL-21 and IL-23 contribute to the formation of pathogenic Th17 cells, while IFN-γ, IL-2 and IL-4 inhibit the process. Evidence supports the participation in a Th17-mediated response in the pathogenesis of pneumonia caused by SARS-CoV-2.
Grifoni et al., 2021 (50)	Review	Review how epitopes identified throughout the SARS-CoV2 proteome reveal a significant correlation between the number of defined epitopes and the size of the antigen provenance and report additional analyzes of SARS-CoV-2 human CD4 and CD8 T cell epitope data.	HLA-restricted epitopes were identified for 30 class I and 45 class II molecules, providing good coverage of a number of different loci and alleles. However, although the average number of epitopes per allele is 15, it ranged from 1 to 219, with a strong bias towards the HLA alleles that are found most frequently in the general population. It was noted that while twenty studies defined class I/CD8 epitopes, only nine defined class II/CD4 epitopes. Furthermore, given the prominent role of CD4+ T responses in SARS-CoV-2 immune responses in the context of natural infection and vaccination. In terms of antigens targeted by epitope identification studies, ten studies tracked peptides derived from the entire proteome, but fifteen studies focused on specific subsets of antigens, primarily based on the fact that the main T cell antigenic targets of SARS-CoV-2 have been independently defined using *pools* of overlapping peptides. Structural proteins (S, M and N) are the dominant targets of T cell responses, but ORF3, ORF8, nsp3, nsp4, and nsp12 are also frequently targets. Within the main antigens, the immunodominant regions are typically pronounced in the case of CD4 recognition, but less so in the case of CD8 responses, which are more evenly distributed among the dominant antigens.
Kalpakci et al., 2020 (51)	Cross-sectional study	Reveal the relationship between T cell subsets and disease severity.	Lymphocyte subsets, CD4+ and CD8+ T cells, memory CD4+ T cells, memory CD8+ T cells, naive CD4+ T cells, effector memory CD4+ T cells, central memory CD4+ T cells, and central memory T cells CD3+, CD4+, CD25+ were significantly lower in critically ill patients. The ratio of *naive T* cells/CD4 + EM T cells was relatively reduced in severe disease. Peripheral CD4 + CD8 + double positive T cells were significantly lower in the presentations of severe disease.
Prompetchara; Ketloy; Palaga, 2020 (52)	Review	Provide a comparative view between SARS-CoV, MERS-CoV, and the new SARS-CoV-2 epidemic in hopes of getting a better understanding of the host-pathogen interaction, host immune responses, and the pathogen immune evasion strategies.	Coronaviruses interfere at several steps during the initial innate immune response, including RNA detection, signaling the type I IFN production pathway, and STAT1/2 activation downstream of IFN/IFNAR, as indicated by suppressor tags. These delayed or attenuating responses of type I IFN interfere with adaptive immune activation. Prolonged viral persistence exacerbates inflammatory responses that can lead to exhaustion and immune suppression as a feedback regulatory mechanism. The biased response of type Th2 also favors the poor outcome of the disease.
Abers et al., 2021 (53)	Cohort study	Analyze the levels of 66 soluble biomarkers in 175 Italian patients with COVID-19 ranging from mild/moderate to critical severity, and we evaluated transcriptional signatures dependent on IFN- type I, IFN- type II, and NF- κ B -dependent whole blood transcriptional signatures.	The disagreement between IFN-α2 protein and IFNA2 blood transcription levels suggests that type I IFNs during COVID-19 may be produced primarily by tissue-resident cells. Multivariate analysis of the first patient samples revealed 12 biomarkers (CCL2, IL-15, soluble ST2 [sST2], NGAL, sTNFRSF1A, ferritin, IL-6, S100A9, MMP-9, IL-2, sVEGFR1, IL-10) that when increased were independently associated with mortality. Multivariate analyzes of longitudinal biomarker trajectories identified eight of the biomarkers mentioned above (IL-15, IL-2, NGAL, CCL2, MMP-9, sTNFRSF1A, sST2, IL-10) and two additional biomarkers (lactoferrin, CXCL9) that were substantially associated with mortality when increased, while IL-1α was associated with mortality when decreased. Among these, sST2, sTNFRSF1A, IL-10, and IL-15 were consistently higher throughout the hospitalization course in patients who died versus those who recovered.
Bunders; Altfeld, 2020 (54)	Review	Understand the underlying mechanisms for the differences between women and male individuals infected with SARS-CoV-2.	Studies in mice have shown that estrogens promote and testosterone can suppress antibody development, and human studies have described lower immune responses to influenza vaccination in men than women, particularly in men with high testosterone levels at the time of vaccination. The ability to induce or maintain antibody responses further declines with age, particularly in men. Sex differences in T cell phenotypes and functions have been reported, including an expansion of Treg cells through estrogen. The FoxP3 gene, which encodes FoxP3, which determines Treg cell differentiation, is expressed on the X chromosome, as is the CD40L gene, which encodes CD40L, which is involved in the CD4 + T cell-mediated help for B cells. Therefore, the increased ability of women’s T cells to reduce excessive immune activation and promote differentiation of B cells may contribute to the less frequent development of severe COVID-19 in women. Recent studies have shown that men have a higher number of Treg than women, but their role in pathogenesis is unclear.
Takahashi et al., 2020 (55)	Review	To describe candidate pathways, ACE2 genetics, and sex hormones as sex differences in COVID-19.	The phenotypes of T cells in all sexes revealed that male patients whose disease worsened had a significantly lower proportion of activated T cells (CD38+HLA-DR+) and terminally differentiated T cells (PD-1+, TIM-3+) and trends for less IFN-γ+ and CD8+ T cells at the first sample collection. However, in women, the deteriorated group had similar levels of these types of CD8+ T cells compared to the stabilized group. The correlation matrix revealed that, in women, higher innate immune system cytokines such as TNFSF10 and IL-15 were positively correlated with disease progression, while there was no association between CD8+ T cell status and deterioration. In particular, CXCL10, M-CSF, and IL-15 were positively correlated with CD8 + IFN-γ + T cells in female patients. On the contrary, in male patients, progressive disease was associated with older age, higher body mass index (BMI), and low activation of CD8+ T cell.
Viveiros et al., 2021 (56)	Review	To describe candidate pathways, ACE2 genetics, and sex hormones as sex differences in COVID-19.	The increase in severity in men is correlated with increased plasma levels of innate immune system cytokines, including IL-8 and IL-18. However, the reduced severity in women corresponds to a greater activation of T cells. Women generally have a greater number of B cells and antibody production. Women have a higher number of CD4+ T cells and proliferation of activated T cells and T cells than men. In COVID-19, women have increased IFN-α secretion and early virus detection and immediate antiviral response after TLR7 stimulation in dendritic cells.
Gao et al., 2020 (32)	*In vitro* assay	Investigate the binding between the N protein of SARS-CoV-2 and MASP-2 and delineate the domains of interaction of the two proteins.	C1 esterase inhibitors (C1-INH) regulate the endogenous coagulation/complement pathway by inhibiting several pathways, including factor XII activation. Deficiency or loss of C1-INH function can lead to hypercoagulation and fibrinolysis. They examined lung samples from patients with SARS-CoV-2 and found a significant increase in macrophage and neutrophil infiltration, both types of cells that express elevated levels of the C5a1 receptor. The analysis of bronchoalveolar lavage fluid (BALF)showed increased levels of IL-6 and the C-X-C motivator ligand 8 (CXCL8), but C5a was detected at concentrations greater than 1000 pg/ml.
Zheng et al., 2020b (57)	Cohort study	To verify whether cytotoxic lymphocytes in patients infected with SARS-CoV-2 become functionally exhausted.	The NKG2A immunological checkpoint increases with reduced ability to produce CD107a, IFN-γ, IL-2, granzyme B and TNF- α on NK cells and CD8+ T cells for disease progression in early stage COVID-19.
Gomez-Lopez et al., 2022 (58)	*In vitro* assay.	To determine cellular immune responses to SARS-CoV-2 particles and proteins/peptides in pregnant women.	In contrast to stricter regulation of traditional Th activities during pregnancy, the regulating element of T cell immunity increases. In both pregnant and nonpregnant women, T cell activation is shown in response to SARS-CoV-2 Ags. Along with a particular reduction in CD24 expression in response to activation by the SARS-CoV-2 particle, a specific increase in CD22 expression was also observed during pregnancy. ‘The peripheral CD23 CD19 + CD20 + B lymphocytes of pregnant and non-pregnant women shrank after stimulation, with the shrankage of pregnant women more severe. Although both research groups had an increase in CD24 + CD38 + CD19 + CD20 + and CD86 + CD80 + CD19 + CD20 + B cells after stimulation, the response was less pronounced in pregnant women than in non-pregnant women. However, protein S alone does not trigger an immune response. Basal levels of certain cytokines (e.g., IFN-, IL-1, IL-18, TRAIL, and IL-27) in mother’s blood are affected by pregnancy status. Furthermore, after being exposed to SARS-CoV-2 particles, maternal mononuclear cells produced fewer IFN- and IL-8, suggesting that pregnancy imprints a distinct cytokine response to coronavirus infection.
Laurén et al., 2022 (59)	Cohort study.	Investigate the correlation between symptomatology and cellular immune responses four to five months after seroconversion based on a specific peptide for severe acute respiratory syndrome coronavirus 2 (SARS-CoV-2).	Regarding SARS-CoV-2 long-term memory specific, T cell responses show dual IFN-γ and IL-2 responses, in contrast to cross-reactive memory T cell responses, which mainly generate IFN-γ in response to stimulation of the SARS-CoV-2 peptide. Persistent humoral immune responses and T cell responses were linked. The severity of the disease of the disease, along with COVID-19-specific symptoms, was correlated with the degree ofSARS-CoV-2-specific memory T cell response four to five months after seroconversion.
Havervall et al., 2022 (60)	Cross-sectional study.	To demonstrate the differences in antibody kinetics depending on the antigen, arguing against the use of the nucleocapsid protein as the target antigen in population-based SARS-CoV-2 serological surveys.	Compared to study healthcare professionals four months after infection, COVID-19 patients who had previously been hospitalized had substantially higher levels of anti-spike and anti-nucleocapsid IgG levels throughout the same period. These results suggest a long-lasting and effective humoral response following SARS-CoV-2 infection, even in the absence or absence of symptoms, even though the size of the humoral response has been linked to the severity of the disease.
Viurcos-Sanabria et al., 2022 (61)	Cohort study.	Examine the *in vitro* response pattern of CD4+ and CD8+ T cells and monocyte subsets to polyclonal stimuli, including the SARS-CoV-2 Spike protein.	High expression of PD-1, low production of IL-2 and IFN-gamma in CD4+ and CD8+ T cells, and poor expression of IFN-alpha in traditional cells and nonclassical monocytes are all related to a baseline immune response profile that is connected to a propensity to develop serious COVID-19. Because antibody titers did not change between moderate and severe instances, these results imply that cellular immunity, rather than humoral immunity, may be more important in stopping the course of COVID-19.
Moore et al., 2022 (62)	Cohort study.	Investigate specific serological antibodies for SARS-CoV-2 by polyclonal *in vitro* reactivation of MBC populations followed by analysis using SAR-CoV-2 antigen-specific B cell ELISpots and IgG antibody ELISAs.	There were few variations in the severity between donor groups, but donors who tested positive for COVID-19 had robust responses that involved antigen-specific memory B cells (MBC). When comparing MBC responses with serological antigen-specific IgG antibody levels, it was shown that MBC responses were significantly sustained six months after recovery from infection.
Moga; Lynton-Pons; Domingo, 2022 (63)	Review	To analyze the critical role of cellular immunity in modulating the development of high-affinity neutralizing antibodies and germinal center B cell differentiation into long-lived, memory-secreting antibody-secreting cells.	The number of circulating Th1 cytokine-producing CXCR5+ Tfh and CXCR5- non-Tfh cells, CD4+ CXCR3+ and CD8+ IFN-γ-producing proliferative memory T cells increased in individuals with moderate symptoms of SARS-CoV-2 infection after a few months. The highest frequency of numerous cytokine production was saw in CD8+ memory T cells specific for SARS-CoV-2 M and N proteins. The production of T-stem cell memory (TSCM) is necessary for long-term memory T-cell success. Convalescent patients with COVID-19 show SARS-CoV-2-specific TSCM cells, suggesting that memory of T cells acquired during SARS-CoV-2 infection may be long-lasting. Furthermore, the existence of SARS-CoV-2-specific central memory T cells that secrete IL-2 and IFN-γ+ IL-2- may indicate long-term memory characteristics. The two subgroups of stem cell-like progenitors of CCR7+ are as follows: CCR7 + PD-1 + TIGIT + cells appear to have depleted properties, CCR7 + PD-1-TIGIT cells have stem cell-like characteristics. Rarely expressing PD-1 and TIGIT, SARS-CoV-2-specific TSCM cells are working memory T cells rather than progenitors that have been impoverished. The lower severity of the illness was related to the presence of IFN-γ CD4+ Th1-producing cells specific for SARS-CoV-2 and CD8+ cytotoxic cells.
Rovito et al., 2022 (64)	Review	Review the current understanding of viral and host factors that contribute to immune dysregulation associated with COVID-19 severity.	There were found to be two primary immunotypes: immunotype 1, connected with specificity and characterized by highly activated CD4 T cells, circulating follicular helper T cells (cTfh) and enhanced CD8 T cells; and immunotype 2, which was much more advantageous and characterized by CD4 T cells and activated T cells, T cells CD4 effector Tbet+, and functional B cells. The B cell categories were distinguished by antibody-producing cells that are CD19 + CD27 + CD38 + and activated naive CD11 + B cells that develop into effector B cells without naive (IgD) and memory markers (CD27), commonly known as double negative B cells, which are CD19 + CD27 - CD38 - CD24 - IgD - CD11c + CD21.
Gurevich et al., 2022 (65)	Cohort study.	Investigate antiviral adaptive immunity to memory B cells (MBC) and memory T cells (MTC) in convalescents with coronavirus 2 of severe acute respiratory syndrome (SARS-CoV-2).	Memory B cells (MBC) and memory T cells (MTC) were found in more than 70% of the convalescents; 20% of them were MBC positive but MTC negative, and 10% were MTC positive but MBC negative. Three months after the first appearance of COVID-19, the highest degree of MBC response was discovered and continued up to seven months after infection. MTC was distinguished by an earlier reaction, whereas MBC was considerably improved 3 months after TFO (time after the beginning of COVID-19). The MTC that secretes IFN-γ, IL-2, and polyfunctional IFN-γ+ IL-2- was considerably elevated 1 month after TFO and remained for up to 7 months. At five months after TFO, IFN-γ-secreting MTC secreting IFN-γ showed a decreased response to of S2 N peptide pool.
Björkander et al., 2022 (66)	Cross-sectional study.	Conduct a population-based study of humoral and cellular immunity to SARS-CoV-2 and explore the characteristics of COVID-19 disease in young adults.	A total of 28.4% of the people had seropositivity, with 18.4% having only one IgM positive result. One in seven seropositive people had no symptoms. Although T cell responses were also saw in 17.2% of seronegative patients, B and T cell memory responses were linked to seropositivity. Although T cell responses did not clearly demonstrate any correlation with the time since the putative illness, the number of IgG-producing B cells did. However, all cellular responses examined, except for S1-specific IL-2, were detectable six months after the supposed illness.

### 3.3 Immunological didactic model of COVID-19

The didactic model for COVID-19 was based on the results of this review for mild and moderate cases and for severe and critical cases in separate figures based on immune variability in host defense against infection in different clinical forms, which was described in the topics below. They were represented in chronological order of events by letters (A-Z). The corresponding references are marked within the image for each biological process in numbers within parentheses.

#### 3.3.1 Adaptive immune response to SARS-CoV-2 infection in mild and moderate cases

**Figure 2:**
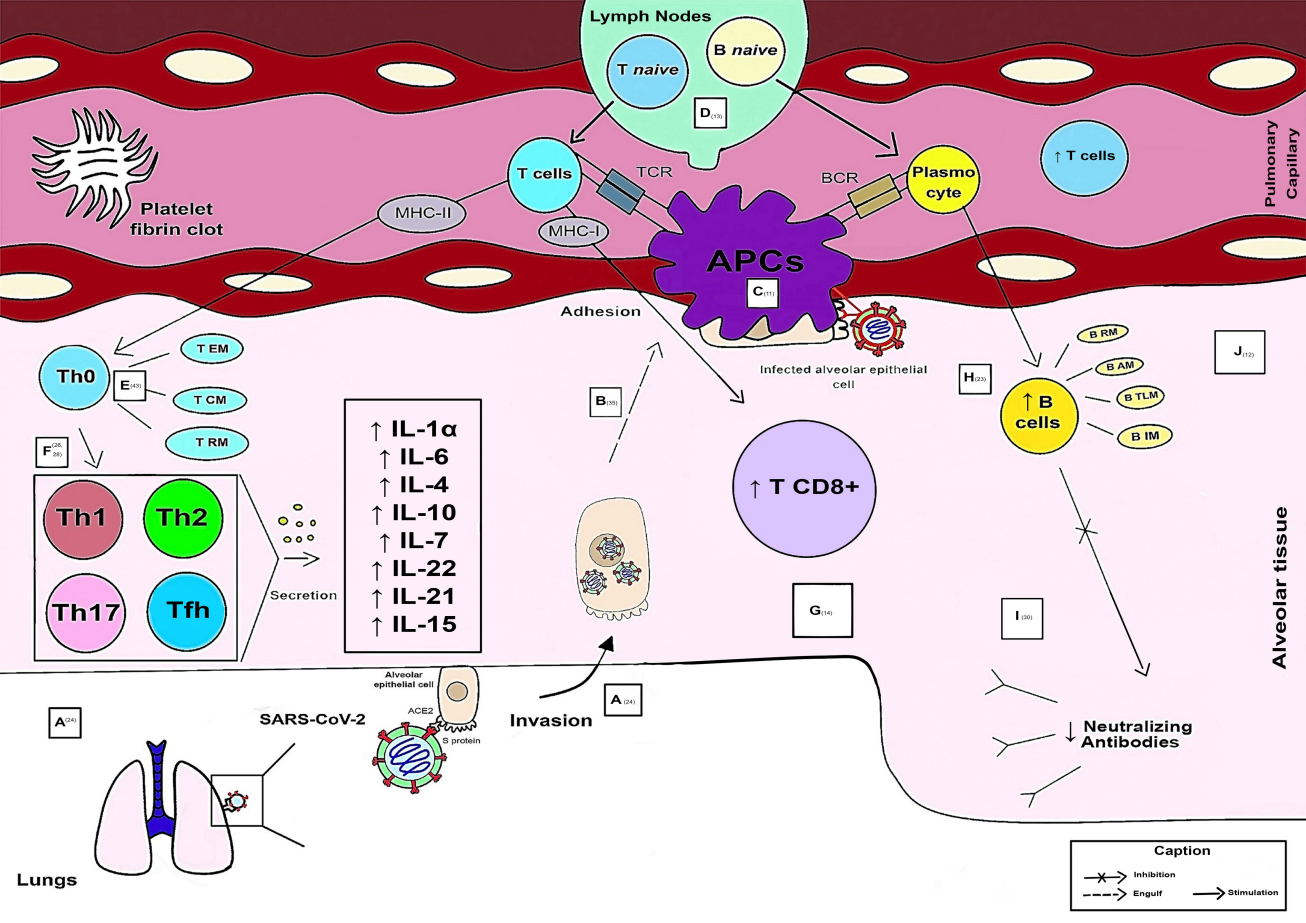
Schematic model of the evolution of the Adaptive Immune Response in mild and moderate cases of COVID-19.

**Figure 2** illustrates adaptive immunity in SARS-CoV-2 infection in mild and moderate cases.

Viral entry into the host occurs through binding of the Spike protein to the human ACE2 receptor in lung cells, mainly alveolar cells rich in this receptor. After activation and activity of the innate response, there was an evolution of the adaptive response (A). The APC is responsible for engulfing the alveolar epithelial cells infected by the virus (during the adhesion process) (B). Viral antigens (Ag) are recognized, processed, and presented through APCs to Ag recognition receptors (T cell receptor - TCR, through MHC-I and MCH-II, and B cell receptor - BCR) of adaptive immune cells (C). When antigens are presented by APCs, lymph nodes activate and secrete naive T and B cells into pulmonary capillaries that travel to the affected alveolar region. Thus, there is a greater release of functional T cells. Coagulopathy generated through platelet fibrin clots may be present in some cases (D). In pulmonary alveolar tissue, CD4+ memory T cells are found (T RM resting memory, T CM central memory and T EM memory effector) (E). The magnitude of T cell responses is maintained in less severe cases. Therefore, from Th0 onward, the responses of the helper T cells are consolidated into subpopulations of Th1, Th2, Th17 and Tfh. The cytokines secreted by these subpopulations of immune cells increase in mild cases and reflect an anti-inflammatory role, such as IL-1α, IL-6, IL-4, IL-10, IL-7, IL-22, IL-21, and IL-15 (F). A larger population of CD8+ T cells specific for SARS-CoV-2 is observed in these patients (G). B cells have a higher count and are subdivided into the following: B RM (resting memory), B AM (activated memory), B IM (intermediate memory), B TLM (tissue-like memory) (H). SARS-CoV-2 neutralizing antibodies levels are decreased in mild and moderate patients are decreased (I). Histopathological conditions caused by the virus, such as edema, fibrosis, hyaline membrane formation, and lymphocytic interstitial infiltration into the alveoli are rarely recorded because of clinical improvement (recovery) and seroconversion (J).

#### 3.3.2 Adaptive immune response to SARS-CoV-2 infection in severe and critical cases

**Figure 3:**
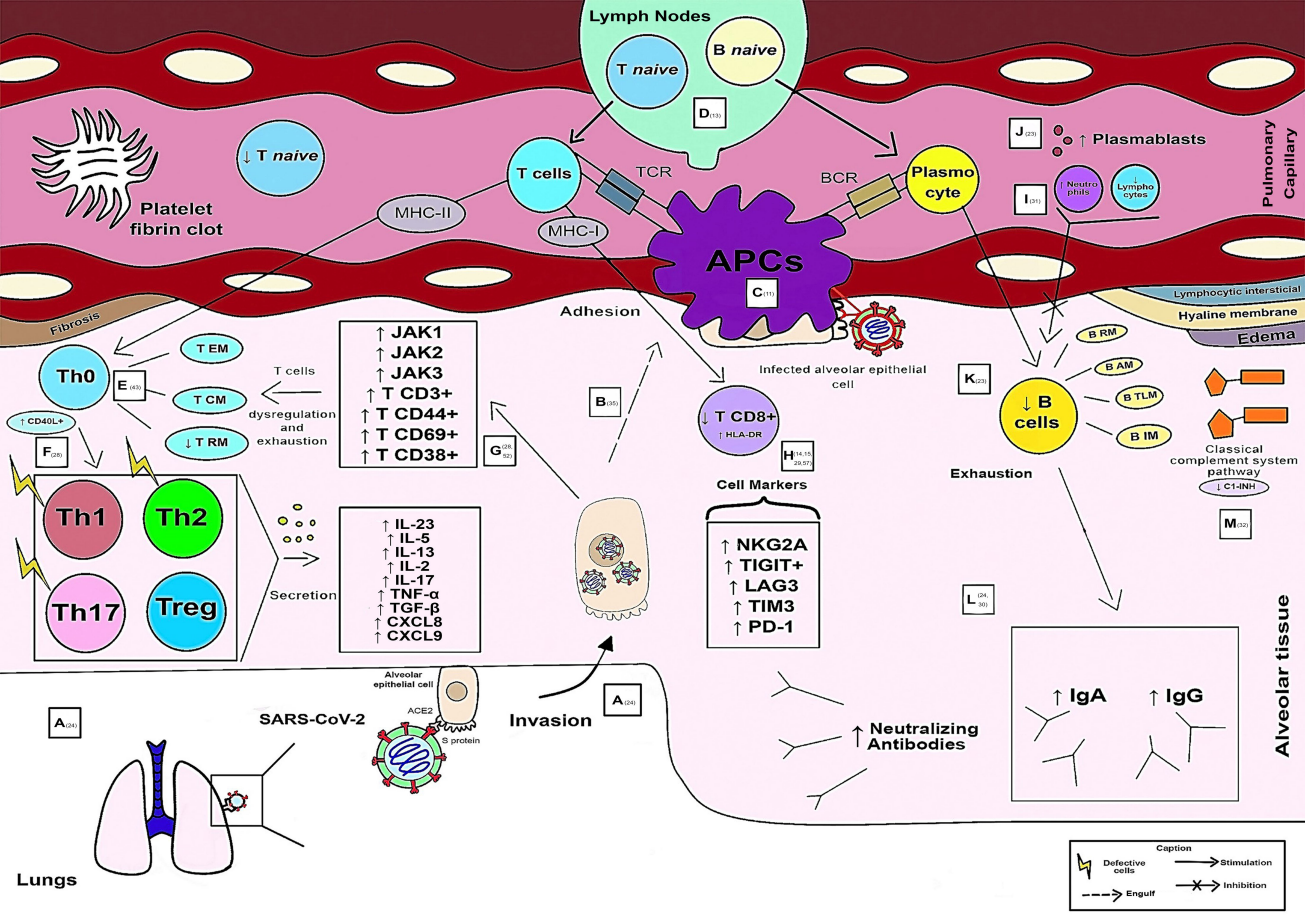
Schematic model of the evolution of the Adaptive Immune Response in severe and critical cases of COVID-19.

**Figure 3** illustrates adaptive immunity in SARS-CoV-2 infection in severe and critical cases.

Viral entry into the host occurs by binding of the Spike protein to the human ACE2 receptor in lung cells, mainly alveolar cells rich in this receptor. After activation and activity of the innate response, there was an evolution of the adaptive response (A). The APC is responsible for engulfing the alveolar epithelial cells infected by the virus (during the adhesion process) (B). Viral antigens (Ag) are recognized, processed, and presented through APCs to Ag recognition receptors (T cell receptor – TCR, through MHC-I and MCH-II, and B cell receptor – BCR) of adaptive immune cells (C). When antigens are presented by APCs, lymph nodes activate and secrete naive T and B cells into pulmonary capillaries that travel to the affected alveolar region. Naive T cells were found at low levels in critically ill patients (D). In pulmonary alveolar tissue, in which CD4+ memory T cells are found (T RM resting memory, T CM central memory and T EM memory effector), there is a functional dysregulation of CD4+ T cells caused by the immunological imbalance of the virus in the individual. Increased expression of CD40L+ in CD4+ T cells (responsible for transmitting signals for cell activation, differentiation, and proliferation) and the low frequency of RM T cells were associated with the severity of the disease (E). From Th0 onward, differentiation and atypical formation of Th cells occur, similar to Th1, Th2, and Th17, but with the absence of main effector immune characteristics and Treg cells. The presence of a subpopulation of Treg cells was associated with the severity of the disease. The cytokines secreted by these subpopulations of immune cells are increased in severe cases and reflect inflammatory conditions, such as IL-23, CXCL8, CXCL9, IL-5, IL-13, IL-17, IL-2, TNF-α, and TGF-β (F). Co-stimulatory molecules such as Janus Kinases (JAK) 1, JAK2, and JAK3 and different types of T cells such as T CD3+, T CD44+, T CD69 +, and T CD38+, stimulated by viral immune dysregulation, are included in severe cases (G). However, CD8+ T lymphocytes are present in fewer numbers, with a high expression rate of human leukocyte antigen (HLA-DR, causing overactivation of these cells) in severe cases of COVID-19. Some markers in CD8+ T lymphocytes were associated with disease severity, such as NKG2A, TIGIT+, TIM3+, lymphocyte activation gene 3 (LAG3+) and programmed cell death protein 1 (PD-1) (H). The ratio of neutrophils to lymphocytes may be a predictor of decreased production of IgM or IgG antibodies and activation and depletion of memory B cells observed during COVID-19, which are positively correlated with the functions of CD4+ T cells (I). Proliferative and metabolically active plasmablasts (plasma cell precursor cells, differentiated B cells capable of producing antibodies) have increased in severe disease (J). B cells are smaller in number and are subdivided into the following: B RM (resting memory), B AM (activated memory), B IM (intermediate memory), B TLM (tissue-like memory) (K). The levels of SARS-CoV-2 neutralizing antibodies in critically ill patients are increased, with persistent and increased levels of IgA and IgG antibodies that have been associated with severe COVID-19 (L). Deficiency or loss of C1 esterase inhibitor function has been observed in hereditary angioneurotic edema (C1-INH) in severe cases that can lead to hypercoagulation (with fibrin clot formation) and fibrinolysis. Additionally, histopathologically, the virus is capable of causing edema, fibrosis, formation of hyaline membranes and lymphocytic interstitial infiltration into the alveoli (M).

## 4 Discussion

Delayed or debilitating type I IFN responses caused by prior dysregulation of the innate immune response disrupt adaptive immune activation. Long-term viral persistence worsens inflammatory reactions, which can fatigue the body and depress the immune system as a regulatory feedback loop (52). In this sense, effective adaptive immunity during viral infections is essential for removing viral particles from the host’s body, preventing the disease from developing to severe stages, and finally recovering afflicted people. Helper T lymphocytes, sometimes referred to as CD4+ T, are, in general, the key players in the adaptive immune response brought about by viral infections. Following virus detection, APCs (antigen-presenting cells) process and deliver viral antigens to CD4+ and CD8+ T lymphocytes. By controlling the course of T cell responses, APCs create a microenvironment of cytokines (15).

In several studies on adaptive immunity and virus, CD4+ and CD8+ T cells were associated with protective response, with greater resolution, despite the uncoordinated responses of these cells and the scarcity of naive T cells, related, among other factors, to aging, seems to make the case more aggravating (19, 35). Compared to antibodies and CD8+ T cells, SARS-CoV-2-specific CD4+ T cells showed a better correlation with lowering the severity of COVID-19 illness. Absolute neutrophil counts and neutrophil-to-lymphocyte ratio may be useful indicators of IgM or IgG antibody response, according to the Rezaei et al., 2021 (31). In this case, the reduction in the number of T cells in patients with severe conditions is negatively related to the production of IL-6 and IL-10 cytokines (11, 25).

The ability of CD4+ T cells to attract additional effector cells to a viral antigen site is one of their main functions. Expression of the CCL3/4/5 gene (MIP-1s) and the CXCL1 chemokine, which is exclusive to CD4+ T cells, may aid in effector cell recruitment. Increased apoptosis and decreased T cell proliferation rates may be the causes of the decrease in T cells in COVID-19, or they may both be contributing factors (25, 40, 63).

Double positive peripheral CD4+ and CD8+ T lymphocytes decreased in cases of illness with severe symptoms (51). A substantial fraction of cTfh is CCR6+, which could indicate accommodation of the mucosal airways. Tfh cells help fight SARS-CoV-2 infection by inducing the generation of neutralizing antibodies (67). The frequencies of cTfh cells (follicular helper T cells or CD4+ T cells) were associated with a reduced severity of the disease. It is complicated and still needs to be better understood how neutralizing antibodies, Tfh cells, and severity, the severity of the disease are related (35).

Specific to circulating S1, CM T cells and T EM (memory effector T cells) have improved effector characteristics, such as activation, proliferation, and production of immune mediators (50). EM T cells are ‘receptionists’ of entry because they are often located in tissues that are easily invaded by pathogenic microorganisms, whereas CM T cells are recruited to secondary lymphoid organs to accelerate the immune response triggered by antigen-presenting dendritic cells. EM T cells and CM T cells with antigen-specific S1 protein activity from most patients have a decreased functional and effector response to at least one parameter, namely activation, proliferation, and secretion of immune mediators (43). Low frequencies of memory T cells (T RM) were found, indicating an impairment of vascular integrity and epithelial barrier function and, therefore, alveolar leakage in critically ill and critically ill patients. Polyclonal T-cell activation is also a risk factor that causes a decrease in the production of certain cytokines together with terminal differentiation in patients (27). SARS-CoV-2 positive B and T cells from working memory were found 12 months after the first natural infection (63).

The presence of virus-specific CD8+ T lymphocytes was associated with improved COVID-19 outcomes in SARS-CoV-2 infection. In addition to that, due to their capacity to eradicate infected cells, CD8+ T cells are essential for recovery from many viral infections. Immune cells (CD8+ T) have programmed cell death protein 1 (PD-1) or differentiation group 279 (CD279) on their surface, which reduce immune system responses, inhibit the activity of inflammatory T cells, and increase self-tolerance. Continuous expression of PD-1 depletes T lymphocytes and reduces their ability to eradicate infectious cells. Although its modes of action are still unclear and may include numerous overlapping processes, CTLA-4 has been shown to be one of the inhibitory immunological checkpoint molecules. By attaching to the CD86 and CD80 ligands, which are similarly engaged in the early excitatory stages of immature and memory T cells, this molecule effectively reduces the excitatory activity of CD28 (15, 68).

COVID-19 is characterized by increased inflammation, which can be generated by regulatory T cells (Tregs). Additionally, FoxP3 induction (Treg cell generator) was considered an important viral pathogenic factor, which is potent in the lungs of patients with severe COVID-19 (25). To discover whether PD-1+ (as observed in patients) in CD8+ T cells in COVID-19 is helpful or leads to the onset of severe illness, more research is required. The Treg-specific transcription factor FoxP3 is well recognized, and T cells that express FoxP3 can inhibit T cell responses. Convalescent patients with COVID-19 had higher levels of FOXP3 expression in circulating CD4+ T cells than healthy subjects. However, in severely ill individuals, the frequency of Treg in the circulation decreases. Critically ill patients in the Intensive Care Unit (ICU) have persistent reductions in all lymphocyte subtypes, especially T cells, while having elevated expression of CD25, CTLA-4 and PD-1in CD4+ T cells. However, FOXP3 expression was not examined (25).

In COVID-19 patients, T cells are expected to exhibit a mixture of Th1, Th2 and Th17 responses, and it is not yet known which Th response is the most protective (25). Regarding the cytokines produced during the adaptive immune response phase in patients with COVID-19, the cytokines IL-2, IL-6, IL-10, IL-4, IL-7, IL-17, and TNF-α were the most cited in disease studies.

IL-2 plays a fundamental role in the dissemination of T cells and in the generation of effector and memory T cells through glucose metabolism, which promotes the proliferation of T, BB and NK cells (69). Thus, this cytokine is crucial for developing of effective immune responses and the preservation of self- tolerance (70). In normal circumstances, IL-2 signaling can activate FOXP3 (Treg cells), which acts as a negative feedback mechanism to stop the IL-2 response. However, in severe COVID-19, this activation is reduced (25). The severity of COVID-19 has a positive correlation with elevated blood levels of IL-2 (27).

IL-6 is a mediator that can inhibit the activation of normal T cells and is therefore the possible cause of lymphopenia in COVID-19 (71). This cytokine is responsible for controlling hematopoiesis, immune response, and inflammation. It may provide regenerative and anti-inflammatory effects by conventional signaling (72), which also suggests these functions during the adaptive phase of the response against infection. IL-6 was the only cytokine among those studied in a study by Lu et al., 2021, which increased in healthy people compared with patients with COVID-19 at this stage of the response to infection (73).

SARS-CoV-2 infection is capable of generating high secretion and activation of cytokines from Th2 cells, among which IL-10 and IL-4 can stimulate an anti-inflammatory role (74). IL-10 is a type 2 cytokine that inhibits the generation of pro-inflammatory cytokines (such as IL-6) in several cell types and acts in immunomodulation. Regarding its role in COVID-19, it has been described to have antagonistic roles, pro-inflammatory and anti-inflammatory, but with positive anti-inflammatory functions in the adaptive phase of the immune response (75). However, IL-4 functions primarily in the activation, multiplication and specialization of B lymphocytes and the production of IgE immunoglobulin, making it a highly sensitive cytokine for the Th2 subset that controls the humoral response (76). Furthermore, IL-4 has been proposed to be related to the metabolic plasticity of different tissues through its anti-inflammatory roles (77). In this sense, IL-4 may be associated with a beneficial role in delaying COVID-19 by inhibiting the inflammatory process (74).

During the disease, patients with severe COVID-19 developed a pattern of increased IL-5, IL-13, eotaxin-2, IgE, and eosinophils (78). IL-5 is a Th2-type cytokine that acts as a specific factor in the maturation and differentiation of eosinophils (79). In patients with COVID-19, it has been associated with a poor prognosis (80). Activated Th2 cells produce IL-13, which acts as a counterregulator of the Th1 immune response, while stimulating the synthesis of TGF-β, which has been correlated with increased severity and greater viral load (80, 81).

Numerous immune cells, including monocytes, macrophages, T cells, and others, can produce TNF-α. This mediator has been associated with pro-inflammatory reactions that are mediated by IL-1 and IL-6 (82). TNF-α, a crucial pleiotropic mediator of acute and chronic systemic inflammatory reactions, has the capacity to control cell proliferation and death, while also enhancing the synthesis of other chemokines and cytokines related, for example, to tissue homeostasis (83). One of the most significant pro-inflammatory cytokines of the innate immune response, TNF-α, can cause Cytokine Release Syndrome (CRS) when its signaling is out of balance. Crosstalk between T cells and other cell types is mediated through connections between responses of TNF and its receptors (TNFR) and regulate positively T cells (84). This cytokine has a positive association with the severity of COVID-19 at this stage of infection associated with hyperinflammatory reactions (cytokine storm) in individuals (15).

IL-7 has a profound impact on lymphocytic differentiation, be it in the development of T cells or plasma homeostasis (necessary for the formation of immature and memory CD4+ T cells, in addition to Th17 cells). It acts on T cell activation, increases the release of other pro-inflammatory cytokines, and correlates negative with TGF-β production (85). These actions depend on their interaction with IL-6 (86). In some life-threatening viral infections, IL-7 treatment restores cell number and functional activity, resulting in lower viral load and clinical improvement (87). In the case of COVID-19, this biomolecule is associated with improved patients outcome with the disease and its inverse relationship with severity, as it allows greater recruitment of T cells (25).

Additionally, through STAT3 activation and the expression of Th17 transcription factors, including ROR-t and AHR, IL-6 signaling promotes Th17 development. Th17 cells produce, for example, the cytokine IL-17, which is elevated in people with autoimmune disorders and inflammatory conditions. It is also generated by CD8+ cells and many subtypes of immature lymphocytes, including gamma delta T cells, NK cells, and group 3 innate lymphoid cells. Therefore, IL-17 is a pro-inflammatory cytokine involved in tissue damage repair, physiological stress reduction, and removal of infection. These roles differ depending on the tissue in which IL-17 is expressed, with the gastrointestinal system and skin being particularly significant. The recruitment of neutrophils by elevated levels of IL-17 in COVID-19 ARDS has been associated with parenchymal damage and lung edema (88).

IL-22 is closely related to tissue repair, especially lung and intestinal epithelial cells, and, during COVID-19, CD4+ T cell activity may effectively participate in lung tissue regeneration. SARS-CoV-2 appears to retain the ability of CD4+ memory T cells to produce IL-22 (27).

The expression of IL-21 has as its main function a robust memory of B cells in COVID-19 (23). Memory B cell activation and exhaustion during COVID-19 are correlated with CD4+ T cell activities. When people have recovered, CD4+ T cells that release IL-21+ and CD4+ T cells that express CD40L+ are better correlated with the affinity formation and maturation of B lymphocytes that are related to protective function (12).

Th17 is important for infections such as COVID-19 and autoimmune. Therefore, in individuals with a dominant Th17 response, medication directed against the Th17 phenotype may be advantageous. Through the inhibition of STAT3, which appears essential for generating the cytokine IL-17A, biological medicines can interfere with cell development toward the Th17 phenotype. Using the Janus kinase (JAK) 2 signaling route, IL-6, IL-23, and IL-21, as well as IL-21 through the JAK1 and JAK3 signaling pathways, activate STAT3. Because IL-23 causes Th17 cells to become pathogenic, inhibiting JAK signaling pathways can stop the growth of pathogenic Th17 cells (23).

IL-15 is a critical mediator for the immunomodulation of inflammation during viral infections. Myeloid cells produce IL-15 to help body T cells react and activate natural killer cells (NK). In mice, IL-15 deficiency promotes airway resistance, as its presence modulates allergen-induced airway obstruction, lowers goblet cell hyperplasia and controls pro-inflammatory cytokines, activating regulatory CD4 T cells, +CD25+Foxp3+ generate IL-10 and IFN-γ. In COVID-19 patients, IL-15 plays a protective role, being more present in mild and moderate cases, in addition to being a possible therapeutic target for the disease (25, 27, 53, 89).

The classical pathway of the complement system is one of the main mechanisms of adaptive humoral immunity. Regarding its activation, there may be an interaction between it and the elevated levels of IL-6 seen in patients with SARS-CoV-2 pneumonia. IL-6 is a potent inducer of C-reactive protein (CRP), capable of initiating complement activation. The initiation of the classical pathway through C1 in patients with severe COVID-19 appears to be impaired, as C1-esterase is low and is related to the thrombotic condition. This is corroborated by individuals with hereditary angioedema (HAE) caused by C1-INH deficiency having higher blood D-dimer levels (90).

Plasmablasts reflect the interactions of adaptive cells with the environment. After seroconversion by the SARS-CoV-2 S protein, plasmablasts lose the type 1 interferon signaling pattern and express gene signatures induced by IL-21 and TGF-β, producing mainly IgG1 and IgA1. In the immune reaction of critically ill patients, there is a change in the programming for inducing IgA2, so that this immunoglobulin does not bind to the dominant antigen of SARS-CoV-2, thus affecting the expression of TGF-β. The cytokine TGF-β is a mediator that regulates angiogenesis, inhibits cell proliferation, and is produced by lymphocytes, macrophages, fibroblasts, smooth muscle cells, chondrocytes, astrocytes, epithelial cells, platelets, and even tumor cells (91).

Notably, the quality of the B-cell response has changed specifically in severely sick ICU patients with COVID-19. These individuals will develop fucosylated IgG antibodies against protein S compared with those with minor symptoms. Fucosylated antibodies improve antibody-dependent cell cytotoxicity and have a higher binding affinity to Fc receptors (ADCC). Future research may help determine whether fucosylated IgG variants activate T cells like their fucosylated counterparts and help decrease the frequency of specific CD4+ T cell antigens seen in ICU patients until the crosslinking of Fc receptors is required for proper cross-presentation of antigen and T-cell initiation (92).

The magnitude and significant elevation of the SARS-CoV-2 peptide-specific neutralizing titers was associated in 2 studies with the severity of the disease, which can serve as a measure of epidemiological control and the evaluation of post-vaccine protective immunity (30, 45). They suggested that a worse result is associated with B-cell activation and proliferation in COVID-19 cases, particularly in severe instances. However, B cells also contribute to antigen presentation and activation of CD4+ T cells (26).

The durability of immunity appears to last 3 to 5 months for SARS-CoV-2 (11, 35, 39). IgA and IgG were shown to be present immediately after the beginning of symptoms and IgA has a stronger effect on viral neutralization than IgG. One month after the beginning of symptoms, serum specific IgA levels decline dramatically, while neutralizing IgA is still measurable in saliva for a longer period (even if it is still very short) (29). IgA levels between hospitalized patients and recovering plasma donors can vary greatly and are not always correlated with standard tests (47).

Regarding gender differences, 4 studies in the review addressed this topic (49, 54–56). The increased severity of COVID-19 in men is related to increased plasma levels of cytokines from the innate immune system (including IL-8 and IL-18). Because T-cell activation depends on the release of sex steroid hormones, the worsening of the male clinical status was associated with a considerably lower percentage of activated T cells (CD38+HLA-DR+), terminally differentiated T cells (PD- +, TIM-3+) and trends toward less IFN-γ+ and CD8+ T cells. In this sense, low testosterone levels in men were associated with a worse clinical outcome (93). Although similar relationships were not observed in female patients, low activation of CD8 + T cells and poor IFN-γ production by CD8 + T cells were strongly related to the age of the patient (55).

There are distinctions between the downstream effects that T cells have on monocytes, depending on the sex. Gender-specific studies revealed that healthy males and male nonventilated patients had higher levels of T-cell-induced up-regulation of CD123 in monocytes than the corresponding groups of female patients. CD123, the α chain of receptors of the interleukin 3, is a surface cell marker present in APCs, such as plasmacytoid dendritic cells (pDCs) and monocytes, critical for activation of adaptive CD4+ and CD8+ T cell responses. Increased binding of T-monocyte cells in males may explain the reported higher plasma concentrations of IL-8 and IL-18 in COVID-19 patients, as IL-3 leads to monocyte mobilization and since high serum levels of unconventional monocytes have been recorded in men, such as CD14+/CD16+, which activate inflammation through the release of these cytokines (94). By encouraging the recruitment of circulating pDCs into the airways and inducing the release of CXCL12 from pulmonary CD123+ epithelial cells, IL-3 boosts innate antiviral immunity (49, 95).

Regarding pregnancy, only 2 studies addressed this and concluded that there is only a small amount of information on the immunological response to COVID-19 throughout this period, postpartum, nursing and in newborns (11, 58). T cell activation is slightly different during pregnancy. After exposure to SARS-CoV-2 particles and the pools of its proteins/peptides, it is also linked to changes in peripheral B cell frequencies and phenotypes (58).

The limitations of this review in terms of its elaboration are: a) the methodology applied (since the search strategy was conducted based on the choice of keywords to answer the main question, so some relevant results may have been missed); b) the results focus on experiments for infection in humans (excluding data on animals with the virus); c) the degree of evidence in which the primary data included were obtained (since the selection of patients and controls came from different criteria and with different sampling and confounding factors); d) differentiation of clinical case definition, as well as severity of the disease in the studies of this review; e) different test methods and assays to investigate the characteristics of adaptive immune cells in the clinical forms evaluated; f) the reviews analyzed in this article present a summarized overview of information (which compromises a more in-depth description of the topic). The limitations also come up against questions not yet answered by the literature, mainly: a) few data found in the literature on the adaptive immune response in pregnant women; b) uncertainty about the durability of humoral responses and their practical significance in relation to sterilizing immunity; c) the durability and protection offered by virus-specific T-cell responses and their relative importance in defending against reinfection compared to antibodies; d) the imprecision of which of the T helper cell responses is/are more resolute.

Despite this, the articles included the present validation and standardization in addition to supporting major contributions to address the guiding question, ensuring an efficient and critical qualitative synthesis of the data. As a result, these factors do not imply significant methodological heterogeneity. These restrictions also highlight the need for immediate research, focusing primarily on postvaccination-scale T cell assays in people to improve the population’s immune surveillance systems and disseminate findings on the interdependence and relative importance of cellular and humoral responses in a balance between protective and pathological responses to make inferences with implications, especially for immunosuppressed and advanced age subgroups.

## 5 Conclusions

Adaptive immunity is diverse, dysregulated, impaired, and delayed in critically ill patients, making it essential to elucidate its immune factors and what makes the clinical outcome different between men and women.

To date, the developed vaccines correspond to great progress in the fight against disease for the formation of herd immunity. Moreover, it was shown here that vaccine production strategies must consider the severity of the clinical case and understanding of protective immunity through ongoing adaptive immunity studies in COVID-19 cases, so that vaccines can induce a long-lasting immunological response against reinfection or possible variants. Due to the limited duration of the antibody response and the emergence of virus variants, it is recommended that vaccines also induce CD4+ and CD8+ T cell responses. The T-cell response is also an essential and necessary component of immunological memory.

## Data availability statement

The original contributions presented in the study are included in the article/supplementary material. Further inquiries can be directed to the corresponding author.

## Author contributions

This document was written by MS and LR. This paper was corrected by KL and LL. All authors contributed to the article and approved the submitted version.

## Conflict of interest

The authors declare that the research was conducted in the absence of any commercial or financial relationships that could be construed as a potential conflict of interest.

## Publisher’s note

All claims expressed in this article are solely those of the authors and do not necessarily represent those of their affiliated organizations, or those of the publisher, the editors and the reviewers. Any product that may be evaluated in this article, or claim that may be made by its manufacturer, is not guaranteed or endorsed by the publisher.
